# On the ozonation of anti-SARS-CoV-2 substances and their nucleoside analogues for mechanistic understanding of the ozone induced transformation using HPLC-ESI-Q-TOF-HRMS

**DOI:** 10.1039/d5ra09800a

**Published:** 2026-03-03

**Authors:** Indra Bartels, Kerstin Hoffmann-Jacobsen, Torsten C. Schmidt, Martin Jaeger

**Affiliations:** a Department of Chemistry and ILOC, Niederrhein University of Applied Sciences Frankenring 20 D-47798 Krefeld Germany martin.jaeger@hs-niederrhein.de; b Faculty of Chemistry, University of Duisburg-Essen Universitätsstraße 5 D-45141 Essen Germany; c Centre for Water and Environmental Research, University of Duisburg-Essen Universitätsstr. 5 45141 Essen Germany

## Abstract

During the COVID-19 pandemic, known and newly developed pharmaceuticals were investigated and approved for treatment against the SARS-CoV-2 virus, among them the prodrug molnupiravir and its active metabolite EIDD-1931. Their reactivity towards ozone is of particular interest, since advanced oxidation processes are heavily researched for their application in wastewater treatment. As a strong oxidant, ozone degrades micropollutants, enhancing contaminant removal. While the ecotoxicological effects of molnupiravir and EIDD-1931 have recently been assessed, the drugs' exposition to ozone has not been studied. In pursuit of the mechanistic elucidation, *N*^4^-hydroxycytosine, which represents a structural subunit of molnupiravir and EIDD-1931, was studied. Further comparison with pyrimidine-2-one and the pyrimidine-based nucleobases cytosine and uracil corroborated the proposed structures of the detected transformation products. The fundamental insights obtained for *N*^4^-hydroxycytosine were then applied to the ozonation of molnupiravir and EIDD-1931. The investigation extended the understanding of the ozonation processes of pyrimidine-2-one derivatives and of this class of antiviral compounds through identification and elucidation of transformation products and through contribution to databases with newly reported MS/MS fragments. While 19 intermediate products and 17 more persistent products were observed, 23 transformation products were assigned identification confidence levels: 2 TPs at level 1, 7 at level 2, 17 at level 3, 4 at level 4, and 6 at level 5. The transformation mechanisms connecting the transformation products were proposed. The findings shall advance the mechanistic understanding of exposing antiviral drugs to ozonation and support the assessment of emerging pharmaceutical micropollutants.

## Abstract 2

HRMS-based mechanistic analysis of the ozonation of molnupiravir and EIDD-1931 reveals key transformation pathways relevant to environmental fate and oxidative wastewater treatment.

## Graphical abstract

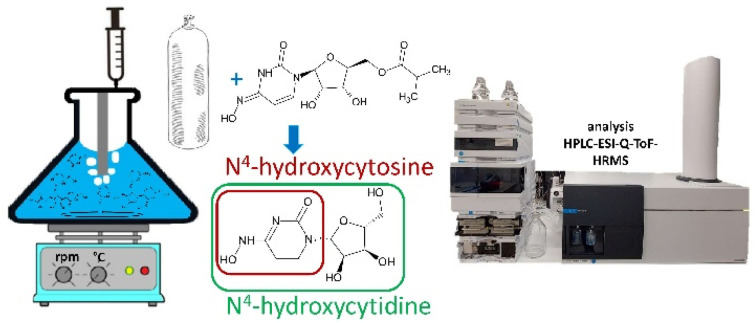

## 1 Introduction

The COVID-19 pandemic, caused by the SARS-CoV-2 virus, necessitated the urgent development and deployment of antiviral agents to mitigate its spread and impact.^2,3^ In addition to micropollutants such as antibiotics or beta-blockers, the concentrations of antivirals in the environment have increased with the rising number of applications and have therefore been recognized as a potential hazard.^4–8^ During the pandemic, the focus was set on antiviral agents that were effective against SARS-CoV-2. Existing drugs, such as favipiravir, remdesivir, and a combination of lopinavir and ritonavir were reevaluated, while other compounds were newly synthesized, *e.g*. molnupiravir (MOL).^9–12^ The pharmaceutical activity in terms of inhibitory concentrations *i.e*., IC_50_ values, is not consistently available for favipiravir, remdesivir, and the combination of lopinavir and ritonavir against SARS-CoV-2 or else the Ebola virus. Available IC_50_ data include 64 µM for favipiravir against Ebola virus and values ranging from 0.010 to 0.069 µM for remdesivir against SARS-CoV-2.^13,14^ For lopinavir, an IC_50_ of 26 µM was determined against SARS-CoV-2, whereas ritonavir showed no detectable inhibitory activity.^15^

Favipiravir has demonstrated efficacy against influenza and Ebola viruses and was approved for clinical use only in Japan, whereas it lacked regulatory authorization in Europe and the USA.^16,17^ Remdesivir shows activity against filoviruses such as Ebola and Marburg virus, as well as coronaviruses beyond SARS-CoV-2 and has received conditional authorization in the EU and is indicated for hospitalized COVID-19 patients.^10,18^ In addition, MOL, originally developed for influenza virus treatment, has shown broad-spectrum antiviral potential against influenza A virus, various seasonal coronaviruses, Ebola virus and hepatitis C virus,^19^ and was therefore administered in the EU between 2022 and 2023 to combat COVID-19.^20^

Even though the COVID-19 pandemic may seem to be over, it inspired continued research into environmental risks and the fate of the described broadband antiviral drugs.^2^

Given the increasing attention to the environmental persistence and transformation of antiviral agents, the European Urban Wastewater Treatment Directive, adopted in 2024, aims to strengthen climate protection and promote water reuse. The directive further highlights the relevance of investigating oxidative degradation pathways of antiviral compounds among others.^21^ Activated carbon adsorption has already been widely implemented as an established advanced treatment for the removal of micropollutants from wastewater.^22^ After intense scientific, technical and economic research, ozonation seems to have established itself as one of the most promising and hence most often realized additional purification stage.^23^ Yet, ozonation as advanced oxidation process (AOP) leads to the formation of transformation products (TPs) resulting from the reaction of ozone or nascent hydroxyl radicals with the parent drugs.^24^ Recent studies have demonstrated that enzymatic post-treatment following ozonation can effectively reduce persistent by-products.^25^ Antiviral compounds and their TPs have been reported together with their biological activities, toxicities, and environmental behavior.^26,27^ However, ozonation of MOL and EIDD has not been investigated yet. Hence, no corresponding TPs have been described. So far, only a single experimental ecotoxicological assessment was conducted for MOL and EIDD,^28^ and an additional *in silico* ecotoxicity prediction using QSAR-based approaches was reported.^29^

The experimental study reported moderate acute toxicity for *Daphnia magna* and *Aliivibrio fischeri*, while the QSAR predictions indicated potential ecological concern. Transformation products formed during oxidative treatment have not been investigated yet, leaving a gap in understanding their fate under advanced wastewater treatment conditions. In this respect, the identification and characterization of TPs are vital for ensuring the safety and effectiveness of ozonation as a purification approach.^30^ Research into the ozonation of anti-SARS-CoV-2 agents is still in its early stages.^31^ For example, the antiviral drug favipiravir used against SARS-CoV-2 could be effectively decomposed using photodegradation, as 99% of the initial concentration of 100 µg L^−1^ was removed within 7 h.^32^ No TPs were identified for favipiravir. Still, all prodrugs were proposed to be first converted into their active metabolites, and second, the active agents were sufficiently stable and exhibited persistence in the environment. During the swine flu epidemic in 2009, the antiviral drug oseltamivir became well known and its oxidation pathway was described.^33^ Reported environmental concentrations of antiviral drugs range from 2 to 482 ng L^−1^, as demonstrated for oseltamivir carboxylate in surface waters and wastewater matrices.^33,34^ For ribavirin, four TPs were identified after treatment with ozone, hydrogen peroxide and peroxymonosulfate.^35^ Studies on favipiravir have reported concentrations of up to 64 µg L^−1^ in effluent samples from wastewater treatment plants (WWTPs), with removal efficiencies of less than 55%.^36^ Additionally, the basic structures of purine derivatives and the virustatic agent acyclovir have previously been used for the investigation of TPs after ozonation.^27,37^ The ozonation of the nucleobases cytosine and uracil, which represent relevant structural subunits of the transformation products discussed below, has been investigated.^38,39^ Hence, a comprehensive mechanistic understanding of the reactions of the drugs and their building blocks is essential to predict the fate of the drugs in aqueous compartments and their potential ecotoxicological impacts, to improve predictions for the environmental degradation of structurally similar compounds, to maximize wastewater treatment efficiency, and to anticipate potential hazards of TPs.^40,41^

The aim of this study was to structurally investigate the degradation and transformation of six compounds chemically related to the antiviral drugs MOL and its active metabolite EIDD-1931 (EIDD) during ozonation. The focus was laid on *N*^4^-hydroxycytosine, followed by the related pyrimidine derivatives cytosine, uracil, and pyrimidine-2-one. From these small model compounds, an understanding of the ozone induced transformations was to be derived. The insights should be extended to the more complex MOL and EIDD. For these purposes, high performance liquid chromatography coupled with high resolution electrospray ionization quadrupole time-of-flight mass spectrometry (HPLC-ESI-Q-TOF-HRMS) was used to monitor and structurally characterize the TPs.^42–44^ Taking advantage of the small molecules as models and the overall structural similarity the ozone induced reaction mechanism was proposed for MOL and EIDD. Using structurally related nucleobases as mechanistic models, this study aims to provide insight into the ozonation of MOL and EIDD. The identification and confidence-based classification of transformation products addresses existing knowledge gaps regarding their ozonation caused fate and supports future investigations of structurally related antiviral pharmaceuticals.

## 2 Materials and methods

### 2.1 Chemicals and reagents

The following antivirals (abbreviation; % purity) were used as listed and given in Table 1 and SI, Table A.1: *N*^4^-hydroxycytosine (*N*^4^-OH-CYT; 97%), cytosine (CYT; 98%), uracil (URA; 98%) and pyrimidine-2-one (PYR; 97%) were obtained from BLD Pharmatech GmbH (Reinbek, Germany). Molnupiravir (MOL; 100%) and EIDD-1931 (EIDD; 99.14%) were received from Cymit Química S.L. (Barcelona, Spain). For dissolving the substances, ultrapure water (Stakpure GmbH, Niederahr, Germany) was used. As eluent A containing 0.1% formic acid (FA, 98–100%; Emsure; Merck KGaA, Darmstadt, Germany) ultrapure water was used for high-performance liquid chromatography (HPLC) and acetonitrile (ACN, ≥ 99.9%; Carl Roth GmbH + Co. KG, Karlsruhe, Germany) was used as eluent B containing 0.1% FA during HPLC measurements.^45^ As a scavenger agent, 10% of tertiary butanol (*tert*-BuOH, >99%, Carl Roth GmbH + Co. KG, Karlsruhe, Germany) was added to the respective test solutions. This 10% solution of *tert*-BuOH corresponded to 1.05 mol L^−1^. The seemingly high quantity as compared to previous studies was chosen because of using continuous ozonation instead of a stock solution.^27^ Hence, in anticipation of potential consumption of *tert*-BuOH, a constant presence of *tert*-BuOH was secured during the entire ozonation period.

**Table 1:** Selected transformation products of *N*^4^-hydroxycytosine, cytosine, uracil, pyrimidin-2-one, molnupiravir, and EIDD-1931 with [M + H]^+^, retention time (*R*_*t*_ in min, in ascending order), and representative MS/MS fragments, reported in descending order of fragment ion intensity. Color coding indicates TPs that are identical to authentic standards: *N*^4^-hydroxycytosine (grey), cytosine (yellow), uracil (red), and pyrimidine-2-one (green). TP [M + H]^+^ = 111.01 (blue) was formed during ozonation of *N*^4^-hydroxycytosine, pyrimidine-2-one, and uracil. For structure proposals see SI Tables A.2–A.7

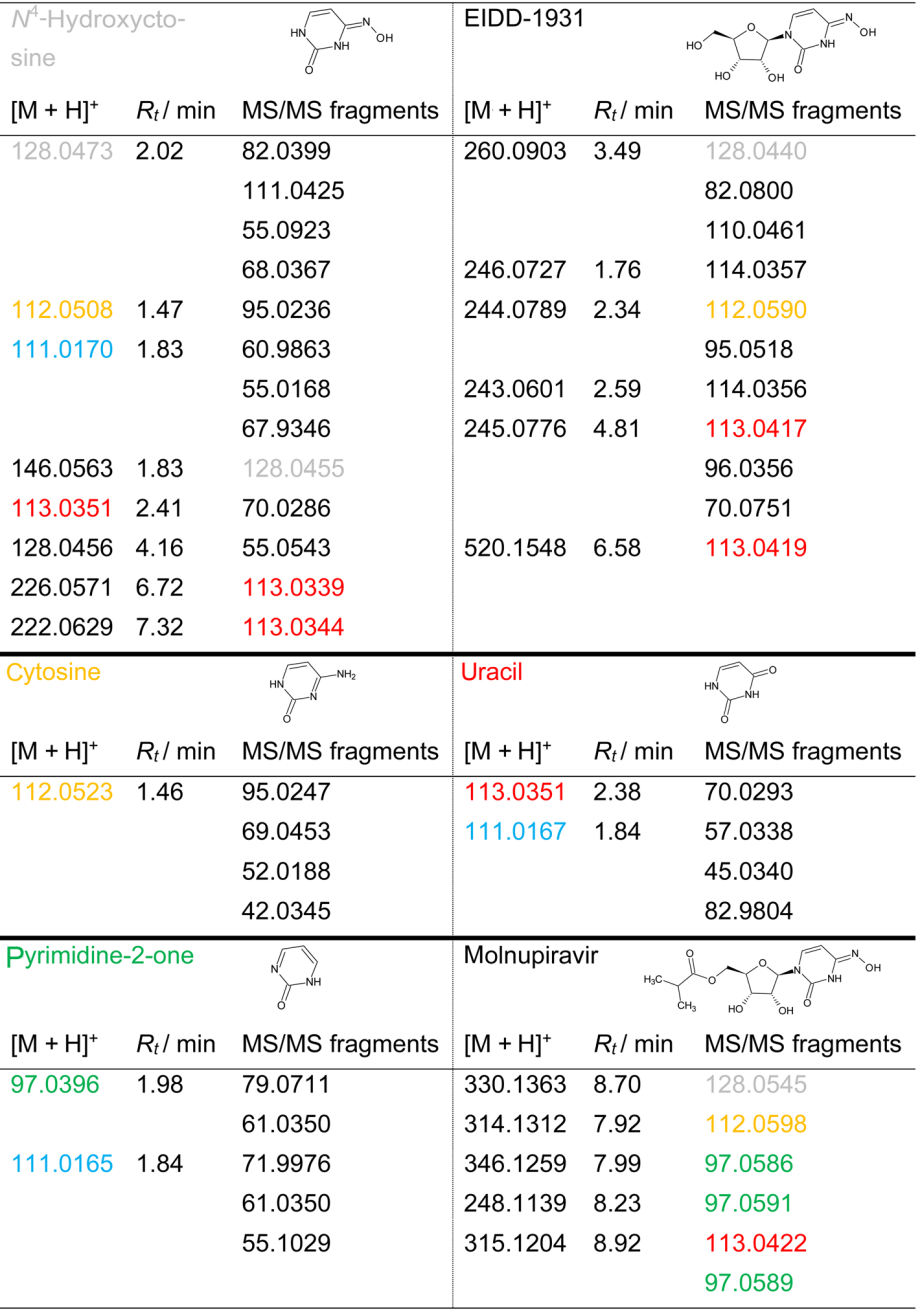

### 2.2 Methods

#### 2.2.1 Experimental setup for ozonation

**Fig. 1:**
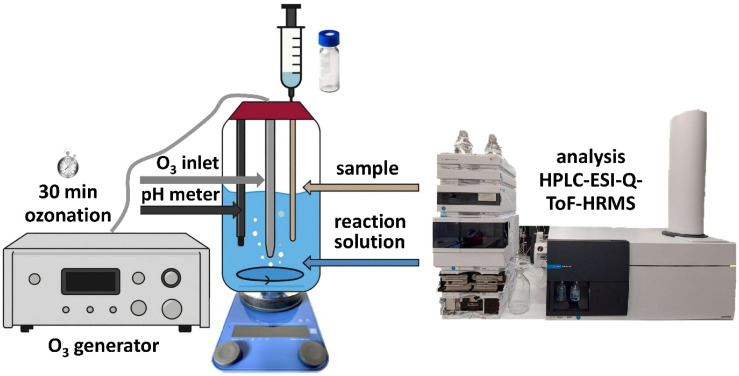
Experimental design for ozonation containing 0.5-L reaction vessel, ozone generator, O_2_ gas bottle, and pH probe with ozone inlet and sampling systems, followed by HPLC-ESI-Q-TOF-HRMS analysis.

In a 500-mL glass vessel equipped with the gas inlet for ozone, 500 mL of the reaction solutions were filled in individually. An ozone generator (COM-AD-02, Anseros, Klaus Nonnenmacher GmbH, Tübingen, Germany) introduced the ozone gas at a controlled concentration of 6.8 g ozone (O_3_)/m^3^. The O_3_ flow rate of 25 L h^−1^ was maintained for 30 minutes during ozonation, yielding an ozone content of 2.8%. In ultrapure water, 20.0 mg L^−1^ of each substance were dissolved and stirred at 300 rpm throughout the ozonation treatment using a magnetic stirrer. At one-minute intervals, 1-mL aliquots were collected from the reaction solution. The collected samples were subsequently purged with compressed air to avoid any continued reactions with ozone, *cf.*Fig. 1. During the ozonation process, the initial pH of 5.5 was found to decrease to 5.0. The temperature was kept at 23.4 °C. The collected samples were analyzed using HPLC-ESI-Q-TOF-HRMS. All ozonation experiments were conducted in compliance with institutional laboratory safety regulations.

For analysis of the degradation reactions, the mass peak areas were plotted as a function of ozonation time. The resulting peak area to reaction time diagrams were evaluated with Matlab (version R2016b, MathWorks Inc.) in normalized dimensions.^45^ Each sample was measured in duplicate. For reasons of graphical clarity, error bars (SD) were omitted. All data, including mean values and standard deviations, are fully available and can be provided upon request.

#### 2.2.2 Quantitative analysis and determination of the transformation products using HPLC-ESI-QTOF-HRMS

A recently published method^45^ was employed for analyzing the compounds qualitatively and quantitatively: an Agilent 1200 HPLC system was equipped with a Dual AJS electrospray ionization (ESI) source and connected to an Agilent 6530 accurate-mass high-resolution (HR) quadrupole time-of-flight (Q-TOF) mass spectrometer (Agilent Technologies, Waldbronn, Germany).

**Fig. 2:**
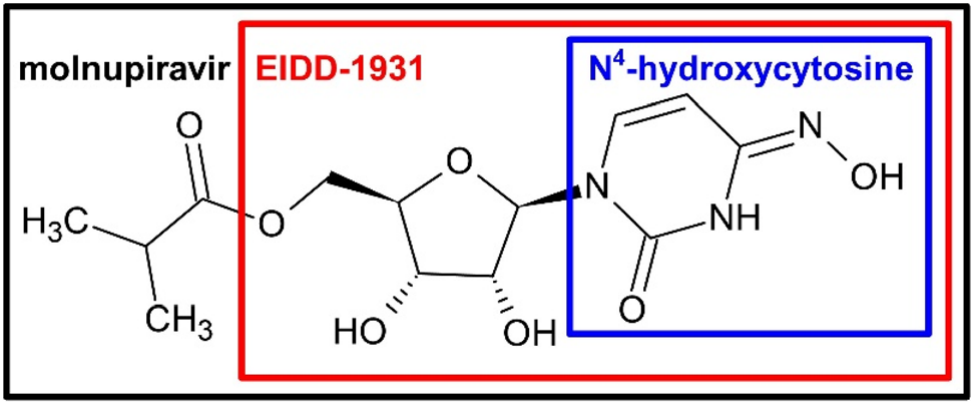
*N* ^4^-Hydroxycytosine moiety as part of EIDD-1931 and the prodrug molnupiravir.

The injection volume was set to 5 µL and the HPLC analysis was performed with the following elution gradient: solvent A (H_2_O + 0.1% FA) and solvent B (ACN + 0.1% FA) were used while adhering to the previously specified conditions, including the choice of column, *i.e*. Zorbax Eclipse C_18_ (Agilent Technologies, Waldbronn, Germany). No modifications were made to the parameters of mass spectrometry (MS) and tandem mass spectrometry (MS/MS), where MS/MS experiments were conducted at 20 eV, as compared to the reported methodology.^45^ System operation and data analysis were managed using MassHunter Workstation B.06.00 software (Agilent Technologies, Waldbronn, Germany). The increasing or decreasing concentrations of each test substance or of their respective TPs, during ozonation period, were plotted as nominal concentrations, which were obtained by dividing the actual concentrations by the initial concentrations. Calibration procedures were applied as previously described. The calibration functions exhibited coefficients of determination (*R*^2^) of 0.9993 for molnupiravir and 0.9991 for EIDD-1931.^45^ Limits of detection (LOD) and limits of quantification (LOQ) were determined based on signal-to-noise ratios of 3 and 10, respectively, under the instrumental conditions described above. The resulting LOD and LOQ values, given as µg L^−1^, were: 13.1, 43.8 for molnupiravir; 1.7, 5.6 for EIDD-1931; 2.3, 7.6 for *N*^4^-hydroxycytosine; 36.4, 121.2 for cytosine; 15.3, 51.1 for uracil; and 37.5, 124.8 for pyrimidine-2-one. Persistence refers to the period of observation, when no further decomposition or transformation of the persistent compound occurred. For the mechanistic interpretation of the subsequently discussed ozonation reactions, the key structural motif shared by the investigated antiviral compounds was considered, *cf.*Fig. 2.

#### 2.2.3 Theoretical aspects of ozonation

During the ozonation process, O_3_ is generated from oxygen gas (O_2_) using a generator. In an aqueous environment, O_3_ undergoes decomposition, resulting in the formation of radicals or directly reacting with dissolved substances (M). This decomposition is pH-dependent, exhibiting an accelerated rate with increasing pH values. Furthermore, the presence of M also induces the decomposition of O_3_ by converting radicals into O_2_.^46^ The reaction model of radical chain reactions starts with initiation. The reaction between O_3_ and hydroxyl radicals leads to the formation of a superoxide anion (˙O_2_^−^) and a hydroperoxyl radical 
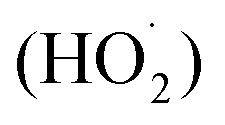
, *cf.*eqn (1):^46^1
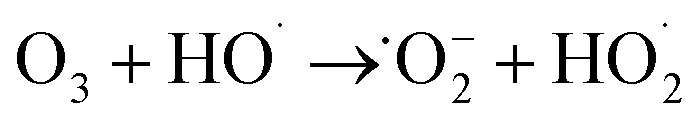
During reaction, an acid-base equilibrium with p*K*_a_ = 4.8 is present. Dissolved substances M react with O_3_ as well. They either react with O_3_ in a direct reaction or form ozonide ion radicals (˙O_3_^−^) by electron transfer. As the reaction continues, ˙O_3_^−^ decomposes by protonation into HO^˙^, which in turn reacts with M. Organic radicals are generated, which add O_2_ and eliminate 
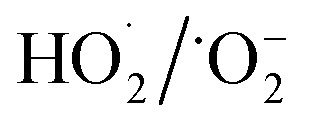
 in a base-catalyzed reaction. The chain termination occurs when M reacts with HO^˙^ or two radicals interact.^46^

The oxidation of olefins proceeds according to Criegee mechanism. First, an unstable cyclic trioxide, the ozonide, is formed. Next, the ozonide is decomposed into a carbonyl compound and hydroxyhydroperoxide. In a final decomposition reaction, a carbonyl compound and hydrogen peroxide are formed from hydroxyhydroperoxide.^47^

## 3 Results and discussion

### 3.1 Comparison of the ozonation products of *N*^4^-hydroxycytosine, cytosine, uracil and pyrimidine-2-one

As key moiety of MOL and EIDD, *cf.*Fig. 2, *N*^4^-hydroxycytosine in solution was exposed to ozone and the transformation products were structurally investigated and concentration-time curves were evaluated.

Ozonation of the parent compound, characterized by the quasimolecular ion [M + H]^+^ = 128.0473, resulted in complete degradation after 15 minutes in pure water, whereas degradation occurred within 7 minutes in 10% aqueous *tert*-BuOH. All other compounds studied degraded slower in the presence of *tert*-BuOH. Since *tert*-BuOH acts as a hydroxyl radical scavenger and hydroxyl radicals possess a higher oxidation potential than ozone, it can be assumed that scavengers suppress the faster radical reaction and hence degradation would proceed more slowly. Therefore, the observed velocity decrease could not be explained conclusively. Ten TPs were observed during the ozonation of *N*^4^-hydroxycytosine, two of them at confidence level 1, *i.e*., compared to a reference standard, one at level 2b, *i.e*., diagnostic evidence, four at level 3, *i.e*., tentative candidates, and three at level 4, *i.e*., molecular formula consistent with MS/MS.^1^ The MS and MS/MS data of cytosine, uracil, pyrimidine-2-one, as well as MOL and EIDD, and their TPs are given in SI Tables A.2 to A.7. Table 1 lists the key transformation products of the analyzed compounds, TPs and their MS/MS fragments, that were observed for more than one parent compound or a TP. Table 1 hence provides a direct comparison of TPs and fragments across the model substances and the drugs.

**Fig. 3:**
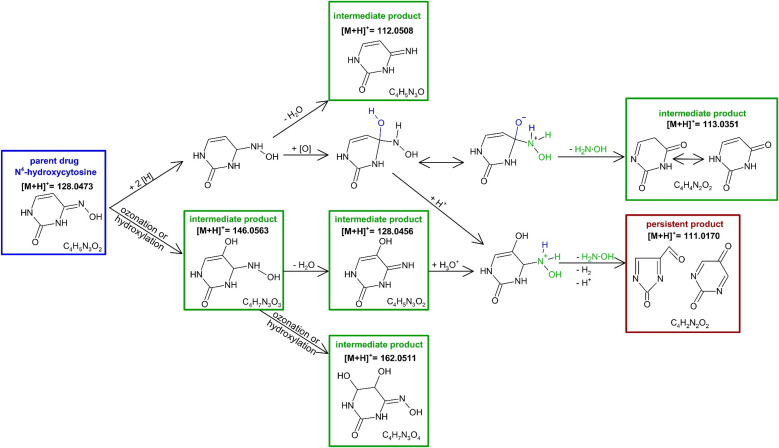
Proposed reaction mechanism of the ozonation of *N*^4^-hydroxycytosine leading *via* the intermediate products to the persistent product *i.e*., whose *c*–*t* curves no longer change during the observation period, *cf.* SI Fig. A.1 blue diamonds.

Ozonation led to the formation of the intermediate TP [M + H]^+^ = 146.0563 *via* ozonation of hydroxylation and exhibited a relatively fast formation, *cf.* SI Fig. A.1. Its MS/MS fragment *m*/*z* = 128.0455 was identified as *N*^4^-hydroxycytosine. Subsequent water elimination from this intermediate product produced TP [M + H]^+^ = 128.0456, *cf.*Fig. 3, which represented an isomer of *N*^4^-hydroxycytosine rather than the parent structure itself, as demonstrated by its MS/MS fragments.

Another intermediate product formed from TP [M + H]^+^ = 146.0563 *via* ozonation or hydroxylation was characterized by an *m*/*z* of 162.0455. It was observed in both deionized water and 10% aqueous *tert*-BuOH. According to the mass-intensity *vs.* time diagram, *cf.* SI Fig. A.1 and Table A.2, this TP appeared relatively late during ozonation. Its retention times and MS/MS fragments resembled those of TPs [M + H]^+^ = 144.0410 and [M + H]^+^ = 147.0398 observed from cytosine and pyrimidine-2-one, respectively.

The recurring TP [M + H]^+^ = 111.0170 was formed through hydroxylamine cleavage. This TP was detected during ozonation of uracil, pyrimidine-2-one, and *N*^4^-hydroxycytosine. Two structural proposals were developed and both were consistent with the observed MS/MS fragments, although neither could be unambiguously assigned. The retention times of these isomers were indistinguishable within experimental uncertainty. Formation of this ion proceeded *via* intermediates [M + H]^+^ = 146.0563 and 128.0456, as illustrated for *N*^4^-hydroxycytosine, *cf.*Fig. 3.

TPs [M + H]^+^ = 112.0508 and 113.0351 were identified as cytosine and uracil, respectively, based on their MS/MS fragments and retention times. These identifications were not available in existing databases but matched the characteristics of the native compounds. TP [M + H]^+^ = 112.0508 formed early during ozonation of *N*^4^-hydroxycytosine. TP [M + H]^+^ = 113.0351 arose from hydroxyl addition, hydroxylamine cleavage, and oxidation, leading to substitution of the imine by an oxo group. This TP remained stable for extended periods, indicating potential persistence under prolonged ozone exposure. Structure proposals for this and the following TPs are listed in SI Table A.2.

TP [M + H]^+^ = 191.0000 was detectable only in the presence of 10% *tert*-BuOH and was observed during EIDD and pyrimidine-2-one ozonation. For TPs [M + H]^+^ = 182.0176, 226.0571, and 222.0629, no molecular structures could be assigned, although MS/MS fragments at *m*/*z* = 113, consistent with uracil-like structures, were observed. Except for TP [M + H]^+^ = 222.0629, all TPs were present at higher concentrations in 10% *tert*-BuOH than in deionized water after 30 minutes of ozonation. TP [M + H]^+^ = 222.0629 fragmented to *m*/*z* = 113.0344, identified as uracil, *cf.* SI Tables A.2 and A.4.

During the ozonation of cytosine, *cf.* SI Fig. A.2, TP [M + H]^+^ = 144.0410 was formed at a later ozonation stage. The MS/MS fragments of the TPs [M + H]^+^ = 116.0462 and 144.0410 showed pronounced differences, where different structures were derived from, *cf.* SI Table A.3 and Fig. A.2. That intermediate underwent two different types of condensation leading to pyrimidinone and 6-amino-2,3-dihydro-4*H*-1,3,5-oxadiazin-4-one.

In the course of ozonation of pyrimidine-2-one, TP [M + H]^+^ = 147.0398 showed a pathway that did not lead to the formation of an oxo-heterocycle, *cf.* SI Fig. A.6, A.7, and Table A.5. Yet, the occurrence of two species with the proposed structures were best accommodated by assuming again a ring opening as intermediate, albeit non-detected product.

TP [M + H]^+^ = 147.0398 of pyridimine-2-one exhibited structural similarity to TP [M + H]^+^ = 144.0410 formed upon the ozonation of cytosine. Differences stemmed from the amino group of cytosine, which did not allow the introduction of a third hydroxy or oxo group.

The combined findings depict the transformation pathways, the intermediate and persistent TPs of the four nucleosides during ozonation. The proposed reaction steps were found consistent with the established concepts of aqueous ozonation.^38,39,46–48^ Direct ozone reactions at electron-rich heterocycles, oxygen incorporation, and cleavages or ring-opening processes have previously been described for pyrimidines and related nucleobases. The interplay between direct ozone reactions and secondary radical chemistry under the applied conditions further followed the general mechanistic framework of aqueous ozonation.

The understanding of the reactions of *N*^4^-hydroxycytosine were most relevant, since the compound constitutes MOL and EIDD. The insights shall hence be taken to the analysis of drug and prodrug.

### 3.2 Ozonation induced reaction pathways of molnupiravir and EIDD-1931 during ozonation: structural insights from basic moiety findings

**Fig. 4:**
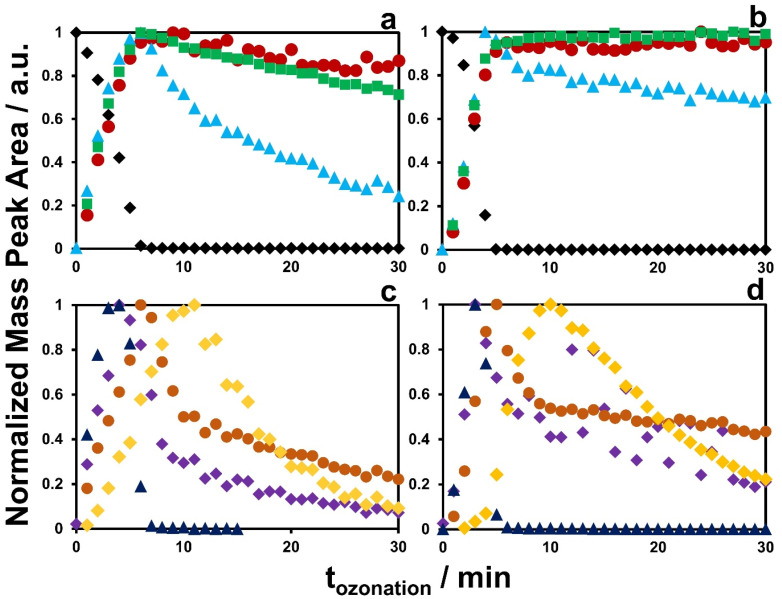
Degradation curves during ozonation of MOL in deionized H_2_O (a and c) and in +10% BuOH (b and d) with parent drug MOL [M + H]^+^ = 330.1363 (black diamonds) and moderately or barely degraded end products [M + H]^+^ = 133.0253 (red circles), [M + H]^+^ = 248.1139 (green squares) and [M + H]^+^ = 346.1259 (blue triangles) in (a) and (b) and intermediate products in (c) and (d) of [M + H]^+^ = 314.1312 (purple diamonds), [M + H]^+^ = 315.1204 (brown circles), [M + H]^+^ = 559.2288 (yellow diamonds) and [M + H]^+^ = 626.2354 (dark blue triangles).

**Fig. 5:**
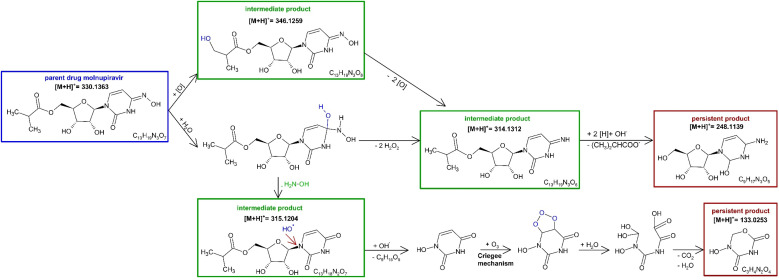
Proposed reaction mechanism of the ozonation of molnupiravir leading *via* the intermediate products to the persistent products *i.e*., whose *c*–*t* curves no longer change during the observation period, *cf.*Fig. 4 green squares and red circles.

**Fig. 6:**
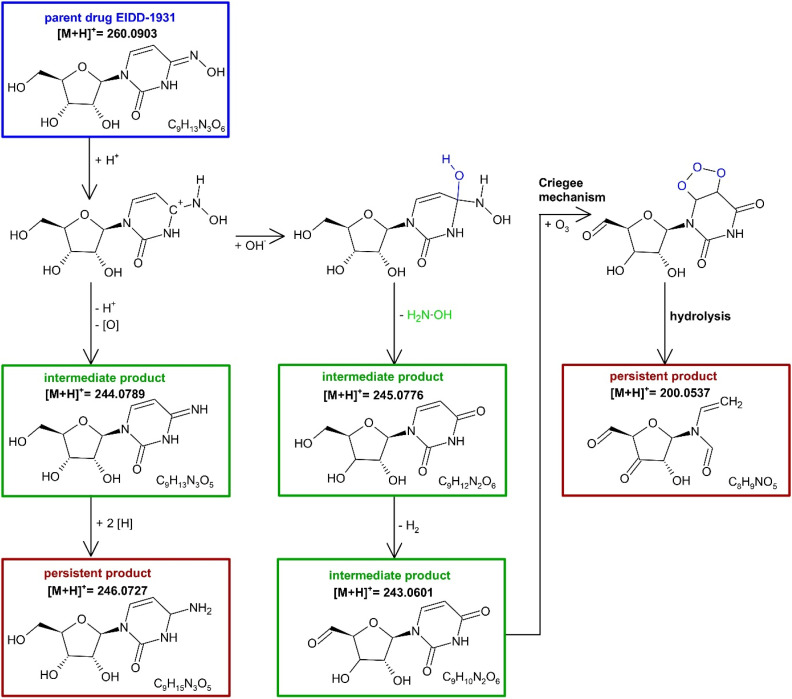
Proposed reaction mechanism of the ozonation of EIDD-1931 leading *via* the intermediate products to the persistent products *i.e*., whose *c*–*t* curves no longer change during the observation period, *cf.* SI Fig. A.8 red squares and gray circles.

The TPs of MOL and EIDD after ozonation in deionized water and in 10% *tert*-BuOH were detected and their presence monitored in terms of mass intensity, *cf.*Fig. 4. The identification was achieved by high-resolution MS and MS/MS. The proposed structures are shown in Fig. 5 and 6. The Criegee mechanism was recognized in several TPs of both compounds, *cf.*Fig. 5 and 6, which was expected since both MOL and EIDD contain a *N*^4^-hydroxycytosine moiety. This connection was evident from the MS/MS fragments of MOL at *m*/*z* = 128.0545 and of EIDD at *m*/*z* = 128.0440, *cf.* SI Tables A.6 and A.7.

The TP of MOL [M + H]^+^ = 248.1139 was formed from the intermediate product with [M + H]^+^ = 314.1312, *cf.*Fig. 4. Its MS/MS fragment at *m*/*z* = 97.0591 indicated a pyrimidine-2-one structural motif. TP [M + H]^+^ = 248.1139, containing a hydroxyl group on the pyrimidine ring, exhibited structural similarity to TP [M + H]^+^ = 246.0727 formed from EIDD, which carries a keto group at the same position. Their structural similarity was verified by the MS/MS fragment with *m*/*z* = 114.0356, *cf.* SI Tables A.6 and A.7. Interestingly, the TPs of MOL with [M + H]^+^ = 315.1204 and [M + H]^+^ = 314.1312 showed a mass difference of *δ* + 1, *cf.*Table 1, and similar *c*–*t* profiles, *cf.*Fig. 4. The same difference applies for cytosine [M + H]^+^ = 112.0523 and uracil [M + H]^+^ = 113.0351. Analysis of MS/MS fragments revealed that TP [M + H]^+^ = 315.1204 yielded a fragment with *m*/*z* = 113.0422, hence as uracil, while TP [M + H]^+^ = 314.1312 generated a fragment ion with *m*/*z* = 112.0598 corresponding to cytosine, *cf.* SI Tables A.3, A.4 and A.6. A further MS/MS fragment ion of TP [M + H]^+^ = 315.1204 was observed at *m*/*z* = 97.0589, indicating a pyrimidine-2-one-related structure, *cf.* SI Tables A.5 and A.6.

As observed for *N*^4^-hydroxycytosine during the formation of TP [M + H]^+^ = 112.0508 and TP [M + H]^+^ = 113.0351, *i.e*., cytosine and uracil, TPs [M + H]^+^ = 314.1312 and 315.1204 displayed comparable *c*–*t* profiles, indicating similar formation kinetics, *cf.*Fig. 4. Furthermore, TP [M + H]^+^ = 314.1312 showed a mass loss of 16 compared to MOL, indicating the elimination of the hydroxyl group from the hydroxylamine, *cf.*Fig. 5. In comparison, TP [M + H]^+^ = 346.1259 exhibited a mass difference of *δ* + 16, suggesting the addition of a hydroxyl group relative to MOL, *i.e.* [M + H]^+^ = 330.1363. However, this addition did not seem to occur at the ring structure, as the fragment *m*/*z* = 97.0586 indicated the presence of the pyrimidine-2-one moiety, *cf.* SI Fig. A.6.

Ozonation of MOL further produced the persistent TP [M + H]^+^ = 133.0253, *cf.*Fig. 4, which was characterized by the molecular formula C_4_H_3_N_2_O_3_, *i.e*. 5-hydroxy-1,3,5-oxadiazinane-2,4-dione. This compound was proposed to be an uracil derivative featuring a hydroxyl substituent as an intermediate. The TP was formed at its highest concentration after 9 minutes of ozonation, but was still observed at the end of ozonation, *cf.*Fig. 4. Hence, TP [M + H]^+^ = 133.0253 was declared to be a persistent product and revealed structural similarities to TP [M + H]^+^ = 116.0462 derived from cytosine, *i.e*. 6-amino-2,3-dihydro-4*H*-1,3,5-oxadiazin-4-one, *cf.*Fig. 5 and SI Fig. A.2. Furthermore, the TP also shared similarities with TP [M + H]^+^ = 112.0508 from *N*^4^-hydroxycytosine *i.e*., cytosine, *cf.*Fig. 3 and SI A.2, and TP [M + H]^+^ = 121.0720 from uracil, *cf.*Fig. 5 and SI A.4. TP [M + H]^+^ = 121.0720 of uracil exhibited a relatively high mass error, *i.e*. *δ* = 92.78 ppm, *cf.* SI Table A.4. Yet, it was considered the most plausible molecular formula based on the number of carbon atoms and compliance with the nitrogen rule. Structural similarities to TP [M + H]^+^ = 117.0294 were apparent, despite of the different number of double bonds, as both TPs contain the 1,3,5-oxadiazinane ring system.

While MOL contains a *N*^4^-hydroxycytosine subunit, the compounds did not show identical transformation products. Only analogous TPs were observed, indicating subtle differences in their oxidative conversion pathways, *cf.*Fig. 4–6.

Nonetheless, the results from structure elucidation indicated mechanistic analogies, particularly the Criegee mechanism, oxygen incorporation, and the generation of cytosine- and uracil-related structures *via* hydroxyl group addition. The correlation between TP [M + H]^+^ = 314.1312 and TP [M + H]^+^ = 315.1204 reflected the cytosine-uracil shift, *i.e*. [M + H]^+^ = 112.0523 and [M + H]^+^ = 113.0351, illustrating the oxidative transformation induced by hydroxyl-radical-driven elimination.

During the ozonation of EIDD, the reaction predominantly occurred at the hydroxylamine group, while the oxolane ring and its hydroxyl substituents proved largely unreactive toward ozone. The absence of shared TPs may be traced back to the unexpected stability of the ester bond towards ozonation, such that the structural motif was retained in several TPs. Only during the pathway to the persistent TP [M + H]^+^ = 133.0253 of MOL, the ester was finally cleaved, *cf.*Fig. 5.

Similar to the reactions observed for MOL, TP [M + H]^+^ = 246.0727 was formed during the ozonation of EIDD from TP [M + H]^+^ = 244.0789. The sequence could be recognized from the *c*–*t* curves, *cf.* SI Fig. A.8. TP [M + H]^+^ = 244.0789 exhibited a mass difference of *δ* − 16 compared to EIDD, indicating the formal loss of an oxygen atom. The resulting MS/MS fragment *i.e.*, *m*/*z* = 112.0590, was identified as cytosine, *cf.*Fig. 6 and SI Table A.7.

The structural similarity between TP [M + H]^+^ = 245.0776 and 243.0601 could likewise be substantiated by the detected fragment at *m*/*z* = 113.0417, indicative of uracil, and the corresponding M^+^ radical cation at *m*/*z* = 114.0356. Although no MS/MS fragment ion at *m*/*z* = 97 was observed, a pyrimidine-2-one-like structure was proposed.

For TP [M + H]^+^ = 559.2288, no molecular structure could be assigned. Since this TP was the last product formed during ozonation, it was presumed to originate from a dimerization process involving [M + H]^+^ = 280, *cf.*Fig. 4. TP [M + H]^+^ = 626.2354, which was formed as the first of the observed transformation products, showed an MS/MS fragment at *m*/*z* = 112.0597, *cf.*Fig. 4 and SI Table A.6, indicating a cytosine constituent. The complete structure could not be satisfactorily elucidated.

The TP with *m*/*z* = 471.1383 during the ozonation of EIDD was interpreted as the dimer of the TP with *m*/*z* = 236. The MS/MS fragment at *m*/*z* = 113.0419 of TP [M + H]^+^ = 520.1548 again was identified as uracil and thus suggested that TP [M + H]^+^ = 520.1548 was a dimer of EIDD-1931 [M + H]^+^ = 260.0903, *cf.* SI Table A.7. No molecular structure could be proposed for TP [M + H]^+^ = 486.1492, either.

The persistent TP [M + H]^+^ = 200.0537, identified as *N*-ethenyl-*N*-[(2*R*,3*S*,5*R*)-5-formyl-3-hydroxy-4-oxooxolan-2-yl]formamide, was the only product observed in this study, which was assigned an open-ring structure formed *via* the Criegee mechanism, *cf.*Fig. 6. Yet, with regard to EIDD, pyrimidine-2-one, and *N*^4^-hydroxycytosine, TP [M + H]^+^ = 191.0000 of *N*^4^-hydroxycytosine was exclusively detected in the presence of 10% *tert*-BuOH, which was attributed to the formation of a BuOH adduct. In contrast, no observable differences occurred for MOL during ozonation in either matrix.

In this study, the identification of key TPs, such as uracil-, cytosine-, and pyrimidine-2-one-related structures, was achieved through accurate MS and MS/MS fragmentation. No qualitative differences were observed between ozonation in deionized water and in 10% *tert*-BuOH. Hence, the proposed structures implied their formation through reactions with ozone or through a combination of ozone and hydroxyl radicals. The slower appearance of TPs in 10% *tert*-BuOH solution supported this hypothesis, as hydroxyl radicals were scavenged, leading to a delayed formation of the TPs, *e.g*. observed during the formation of [M + H]^+^ = 346.1259, *cf.*Fig. 4 and [M + H]^+^ = 520.1548, *cf.* SI Fig. A.8. The structural and kinetic analysis of the smaller nucleotides yielded the foundation for the investigation of MOL and EIDD. The transformation pathways were constructed with the TPs as anchor points. The contribution of the Criegee mechanism was identified and the combination of ozone- and hydroxyl radical-mediated reactions were recognized during the formation of some persistent TPs. Due to the oxolane and ester moieties, MOL and EIDD yielded TPs different from the model compounds. Yet, with respect to the *N*^4^-hydroxycytosine, TPs, reaction pathways and mechanistic considerations were found analogous to the nucleosides.

Similar transformation behavior has been reported for antiviral nucleoside analogues during oxidative water treatment, where structurally related base modifications and fragment motifs were identified.^27,31,48^ The analogy of the transformation further supported the pathways derived for MOL and EIDD.

A comparative overview of previously published ozonation studies on other antiviral pharmaceuticals and related structural motifs is provided in SI Table A.8. These studies report on oseltamivir, acyclovir, and favipiravir using LC-MS or MS/MS, with TPs examined under ozonation conditions, although only few analytes were on purine motifs, *e.g*., adenine and guanine. The current work specifically addresses SARS-CoV-2-related compounds and investigates the ozonation of molnupiravir and EIDD and their transformation products. As can be seen from the literature overview, liquid chromatography together with high-resolution mass spectrometry has become the standard approach.

While the instrumentation is associated with higher costs, it enables non-target screening, accurate mass determination, and MS/MS-based structural elucidation of unknown transformation products. Such capabilities are essential for mechanistic interpretation and confidence-based structure assignment. Future work may focus on transferring identified transformation products into targeted HPLC-MS/MS monitoring strategies or combining HRMS-based screening with bioanalytical approaches for routine environmental assessment.

## 4 Conclusion

The investigation of MOL, EIDD and its nucleoside moieties yielded the identification and concentration-time trends of 36 TPs during ozonation. While ozonation led to severe alterations of the heterocycles in the nucleosides, reactions proceeded at the hydroxylamine moiety in the drug and prodrug. The recognized differences and similarities were predominantly identified by MS/MS fragment comparison with the help of the nucleosides. The radical scavenger *tert*-BuOH exercised weak and non-uniform impact on the reaction suggesting that hydroxyl radical contribution was of minor importance.

Although no identical TPs were detected for the ozonation of MOL and EIDD, analyzing the ozonation and MS/MS fragmentation of the pyrimidine derivatives was essential to fully elucidate the transformation products of MOL and EIDD and their reaction pathways. These analyses provided a fundamental molecular-level understanding of the ozonation processes and the corresponding mechanistic pathways. The applied HPLC-ESI-Q-TOF-HRMS approach proved to be a robust analytical strategy, as mechanistic interpretations were consistently supported by MS/MS fragmentation patterns and confidence-based structure assignments. While HRMS requires advanced instrumentation, it represents a widely established technique in environmental analysis and enables comprehensive structural elucidation without additional experiments.

Insights gained from laboratory studies on the behavior of these compounds in the presence of ozone may help guide the design and optimization of advanced purification strategies relevant to wastewater treatment plants. Moreover, such insights are essential for addressing ecological and human health considerations in modern water management. The occurrence of persistent transformation products suggests that ozonation may benefit from subsequent post-treatment steps, such as biologically active or enzymatic filtration, to further reduce residual transformation products in advanced wastewater treatment systems.^25^ Additionally, the observed persistent TPs emphasize the need to study ecological implications, *i.e*. ecotoxicological risks and the fate of various compounds, as has been demonstrated for the parent drugs.^28^ Experimental ecotoxicological assessment of individual transformation products would require reference standards. This study contributed to the elucidation of the ozonation induced fate of MOL and EIDD and establishes a mechanistic basis for future investigations of related antiviral pharmaceuticals.

## Author contributions

Indra Bartels: conceptualization, methodology, validation, formal analysis, investigation, data curation, writing—original draft, writing—review and editing, visualization, project administration. Kerstin Hoffmann-Jacobsen: writing—review and editing. Torsten C. Schmidt: writing—review and editing, supervision. Martin Jaeger: writing—review and editing, supervision, funding acquisition.

## Conflicts of interest

There are no competing interests to declare.

## Supplementary Material

RA-016-D5RA09800A-s001

## Data Availability

The data supporting this article have been included as part of the supplementary information (SI). Supplementary information: Tables A.1 to A.7, Fig. A.1 to A.8. See DOI: https://doi.org/10.1039/d5ra09800a.
