# Supplementary material for: On the ozonation of anti-SARS-CoV-2 substances and their nucleoside analogues for mechanistic understanding of the ozone induced transformation using HPLC-ESI-Q-TOF-HRMS
Source: RSC Adv. 2026 Mar 3;16(13):12068–79. doi: 10.1039/d5ra09800a (PMC12955708; doi:10.1039/d5ra09800a)
Supplement: RA-016-D5RA09800A-s001 [file RA-016-D5RA09800A-s001.pdf]

Supplementary Information (SI) for RSC Advances.
This journal is © The Royal Society of Chemistry 2026

**RSC Advances**

Supplementary information

# On the ozonation of anti-SARS-CoV-2 substances and their nucleoside analogues for mechanistic understanding of the ozone induced transformation using HPLC-ESI-Q-TOF-HRMS

**Indra Bartels [a,b], Kerstin Hoffmann-Jacobsen [a], Torsten C. Schmidt [b,c], Martin Jaeger [a,*]**

[a] Department of Chemistry and ILOC, Niederrhein University of Applied Sciences, Frankenring 20, D-47798 Krefeld, Germany; [b] Faculty of Chemistry, University Duisburg-Essen, Universitätsstraße 5, D-45141 Essen, Germany; [c] Centre for Water and Environmental Research, University of Duisburg-Essen, Universitätsstr. 5, 45141 Essen, Germany; [*] corresponding author: martin.jaeger@hs-niederrhein.de, Frankenring 20, 47798 Krefeld, Germany

**Table A. 1: Substances measured in positive mode, their structural and molecular formula, as well as the exact and accurate mass of the quasi molecular ion $[M+H]^+$ and the relative mass accuracy *δm/m* in ppm.**

| substance | structure | molecular formula | $[M+H]^+$ exact | accurate | *δm/m* / ppm |
|---|---|---|---|---|---|
| $N^4$-hydroxycytosine | 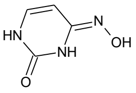 | $C_4H_5N_3O_2$ | 128.0455 | 128.0476 | 16.40 |
| cytosine | 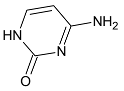 | $C_4H_5N_3O$ | 112.0505 | 112.0524 | 16.96 |
| uracil | 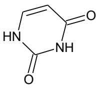 | $C_4H_4N_2O_2$ | 113.0346 | 113.0351 | 4.42 |
| pyrimidine-2-one | 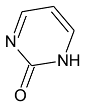 | $C_4H_4N_2O$ | 97.0396 | 97.0399 | 3.09 |
| molnupiravir | 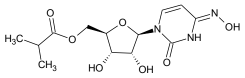 | $C_{13}H_{19}N_3O_7$ | 330.1296 | 330.1361 | 19.69 |
| EIDD-1931 | 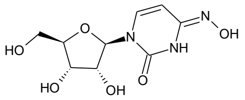 | $C_9H_{13}N_3O_6$ | 260.0877 | 260.0766 | 42.68 |

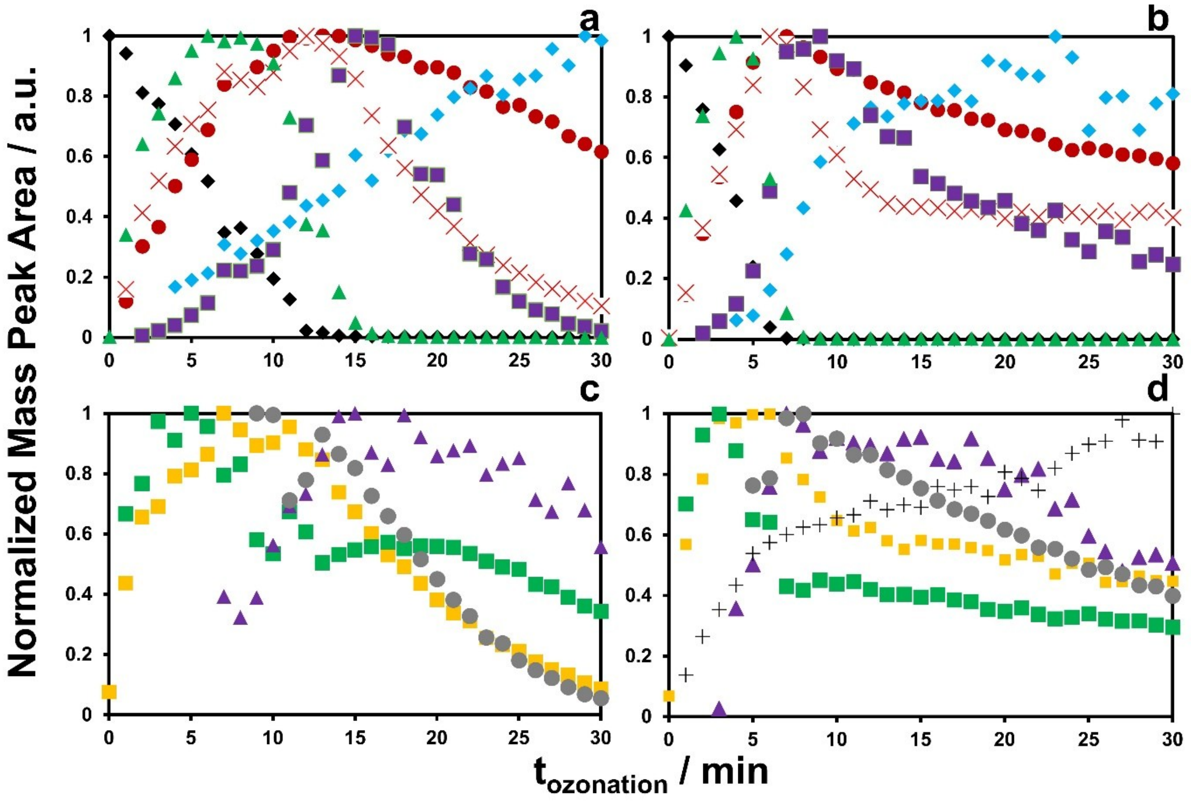


**Figure A. 1: Degradation curves during ozonation of N[4]-hydroxycytosine in deionized $H_2O$ (a) and in +10% BuOH (b) with parent drug N[4]-hydroxycytosine $[M+H]^+$= 128.0473 (black diamonds), and TPs $[M+H]^+$= 162.0511 (red circles), $[M+H]^+$= 111.0170 (blue diamonds), $[M+H]^+$= 113.0351 (red crosses), $[M+H]^+$= 226.0571 (purple squares), $[M+H]^+$= 222.0629 (green triangles) (a,b), and TPs $[M+H]^+$= 182.0176 (purple triangles), $[M+H]^+$= 128.0456 (gray circles), $[M+H]^+$= 112.0508 (yellow squares), $[M+H]^+$= 146.0563 (green squares) (c,d), and TP $[M+H]^+$= 191.0000 (black crosses) (d).**

**Table A. 2: N⁴-hydroxycytosine ([M+H]⁺= 128.0473) and its observed transformation products (TPs) formed during ozonation including retention time (*R*t / min), MS/MS fragmentation patterns, proposed molecular structures, and mass accuracy (*δm/m* / ppm). TPs without proposed structures were marked as not available (n.a.), as more investigations should be necessary for clarification. *m/z* values without proposed fragment structures are shown in parentheses.**

| $[M+H]^+$ | $R_t$ / min | proposed structure | MS/MS fragments | *δm/m* / ppm |
|---|---|---|---|---|
| 128.0473 | 2.015 | 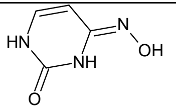 | $[M+H]^+$= 82.0399<br>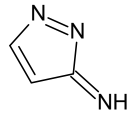<br>$M^{*+}$= 111.0425<br>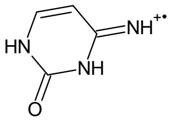<br>$M^+$= 55.0923<br>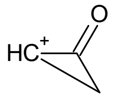<br>$M^{*+}$= 68.0367<br>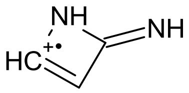 | 14.84 |
| 162.0511 | 1.262 | 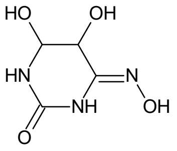 | (56.0135)<br>$[M+H]^+$= 73.0393<br>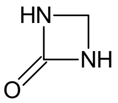<br>$[M+H]^+$= 119.0443<br>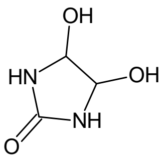<br>$[M+H]^+$= 101.0335<br>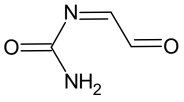 | 1.04 |

**Table A. 2 continued**

| $[M+H]^+$ | $R_t$ / min | proposed structure | MS/MS fragments | $\delta m/m$ / ppm |
|---|---|---|---|---|
| 112.0508 | 1.469 | 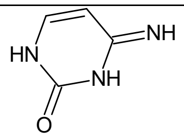 | $M^+$= 95.0236 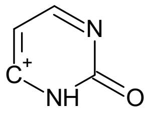 $M^+$= 71.0240 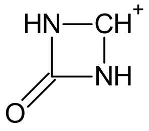 $M^{*+}$= 87.0182 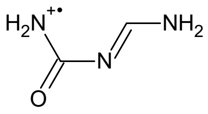 | 2.68 |
| 111.0170 | 1.833 | 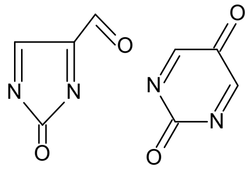 | $M^+$= 55.0168 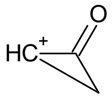 (67.9346) | 17.11 |

**Table A. 2 continued**

| $[M+H]^+$ | $R_t$ / min | proposed structure | MS/MS fragments | *δm/m* / ppm |
|---|---|---|---|---|
| 146.0563 | 1.833 | 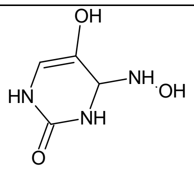 | $[M+H]^+$= 103.0496<br>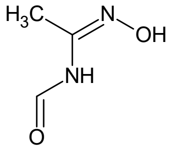<br>(57.0449)<br>$[M+H]^+$= 85.0398<br>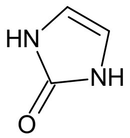<br>$[M+H]^+$= 112.9547<br>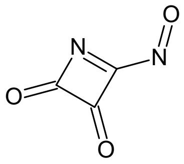<br>$[M+H]^+$= 128.0455<br>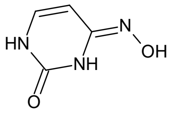 | 2.05 |

39 **Table A. 2 continued**

| $[M+H]^+$ | $R_t$ / min | proposed structure | MS/MS fragments | $\delta m/m$ / ppm |
|---|---|---|---|---|
| 182.0176 | 2.042 | n.a. | (97.9681)<br>(56.9420) | n.a. |
| 113.0351 | 2.407 | 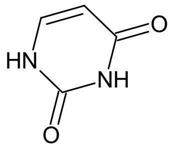 | $M^+$= 70.0286<br>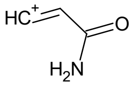<br>$M^+$= 96.0079<br>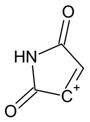 | 4.83 |
| 191.0000 | 3.523 | 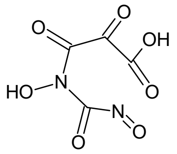 | (84.9589, 129.0001, 56.9414) | 34.15 |
| 128.0456 | 4.156 | 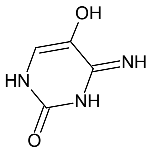 | $M^+$= 55.0543<br>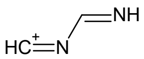<br>(72.9367)<br>(112.1006) | 1.15 |
| 226.0571 | 6.721 | n.a. | $[M+H]^+$= 113.0339<br>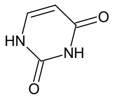<br>(70.0294)<br>(96.0082)<br>(138.9638) | n.a. |
| 222.0629 | 7.321 | n.a. | $[M+H]^+$= 113.0344<br>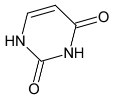 | n.a. |

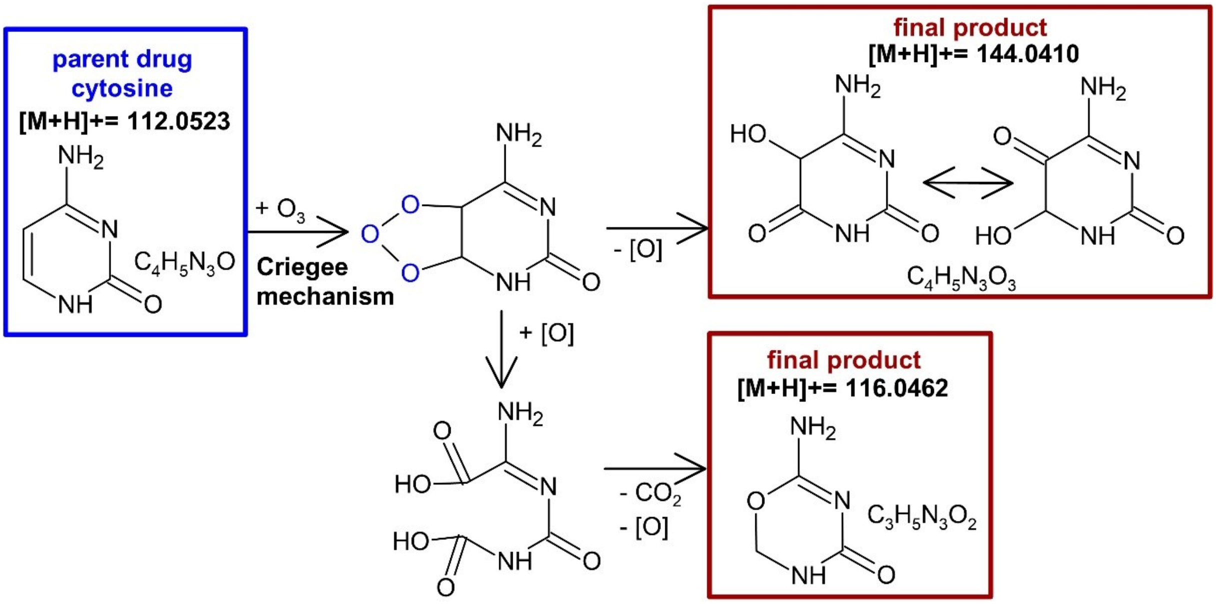


**Figure A. 2: Proposed reaction mechanism of the ozonation of cytosine leading via the intermediate products to the final product *i.e.*, whose *c-t* curves no longer change during the observation period, *cf.* Figure A. 3 red circles and blue squares.**

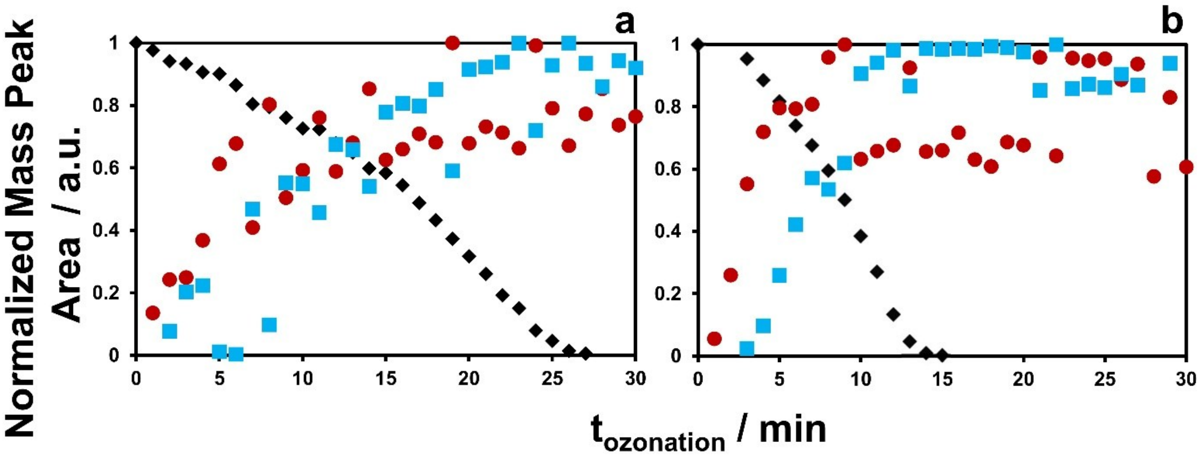


**Figure A. 3: Degradation curves during ozonation of cytosine in deionized $H_2O$ (a) and in +10% BuOH (b) with parent drug cytosine $[M+H]^+$= 112.0523 (black diamonds), and TPs $[M+H]^+$= 116.0462 (red circles) and $[M+H]^+$= 144.0410 (blue squares).**

**Table A. 3: Cytosine ([M+H]$^+$= 112.0523) and its observed transformation products (TPs) formed during ozonation including retention time (*$R_t$* / min), MS/MS fragmentation patterns, proposed molecular structures, and mass accuracy (*δm/m* / ppm).**

| [M+H]$^+$ | *$R_t$* / min | proposed structure | MS/MS fragments | *δm/m* / ppm |
|---|---|---|---|---|
| 112.0523 | 1.463 | 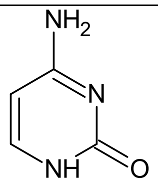 | M$^+$= 95.0247 | 16.06 |
| | | | 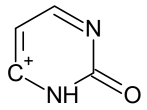 | |
| | | | M$^+$= 69.0453 | |
| | | | 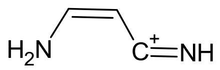 | |
| | | | M$^+$= 52.0188 | |
| | | | 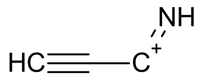 | |
| | | | M$^+$= 42.0345 | |
| | | | 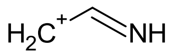 | |
| 116.0462 | 1.245 | 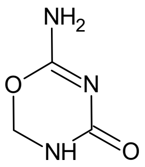 | M$^+$= 55.0296 | 6.44 |
| | | | 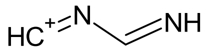 | |
| | | | [M+H]$^+$= 98.0351 | |
| | | | 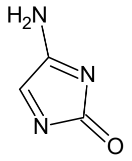 | |

**Table A. 3 continued**

| $[M+H]^+$ | $R_t$ / min | proposed structure | MS/MS fragments | *δm/m* / ppm |
|---|---|---|---|---|
| 116.0462 | 1.245 | 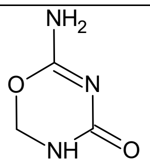 | $[M+H]^+$= 73.0396 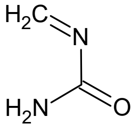 | 6.44 |
| 144.0410 | 1.931 | 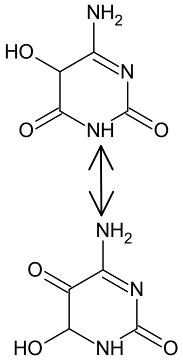 | $[M+H]^+$= 73.0399 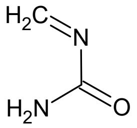 $[M+H]^+$= 44.0137 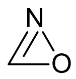 $[M+H]^+$= 99.0194 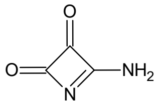 | 4.17 |

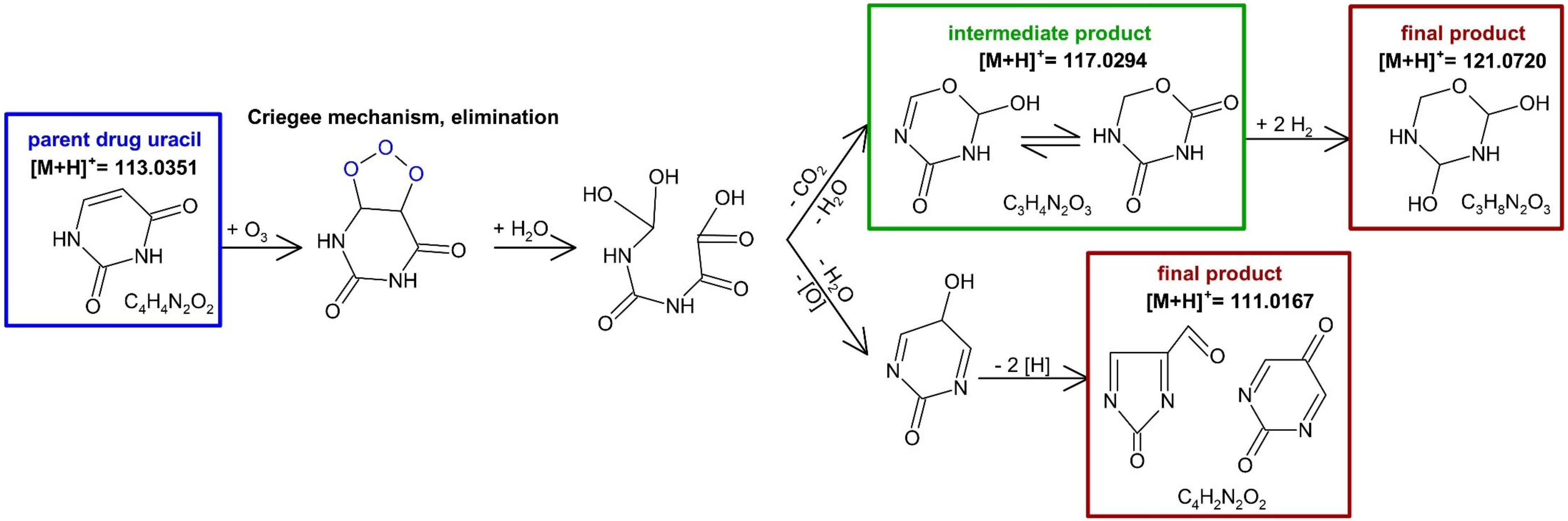


**Figure A. 4: Proposed reaction mechanism of the ozonation of uracil leading via the intermediate products to the final products *i.e.*, whose *c-t* curves no longer change during the observation period, *cf*. Figure A. 5 yellow squares and purple triangles.**

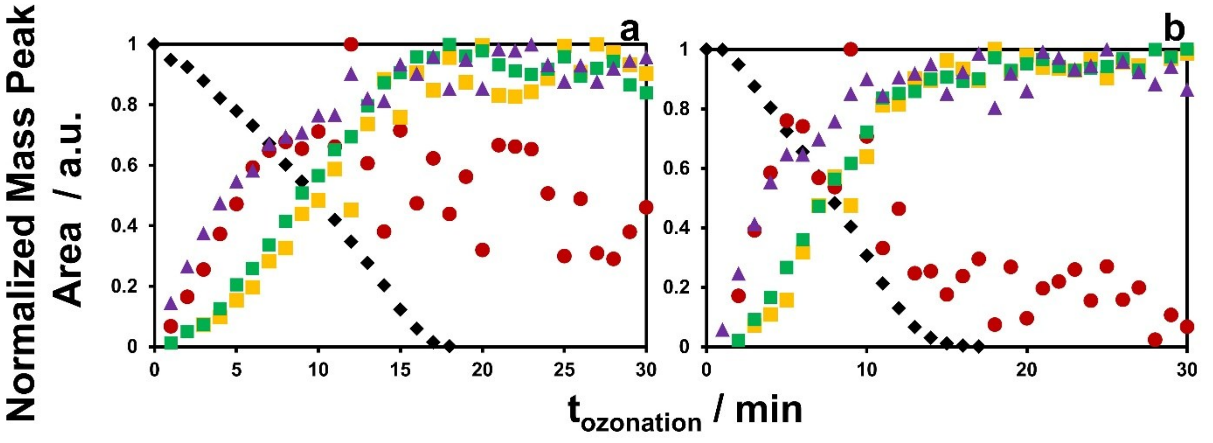


**Figure A. 5: Degradation curves during ozonation of uracil in deionized $H_2O$ (a) and in +10% BuOH (b) with parent drug uracil $[M+H]^+$= 113.0351 (black diamonds), and TPs $[M+H]^+$= 117.0293 (red circles), $[M+H]^+$= 121.0720 (yellow squares), $[M+H]^+$= 293.0311 (green squares) and $[M+H]^+$= 111.0167 (purple triangles).**

**Table A. 4: Uracil ([M+H]$^+$= 113.0351) and its observed transformation products (TPs) formed during ozonation including retention time (*$R_t$* / min), MS/MS fragmentation patterns, proposed molecular structures, and mass accuracy (*δm/m* / ppm). TPs without proposed structures were marked as not available (n.a.), as more investigations should be necessary for clarification. *m/z* values without proposed fragment structures are shown in parentheses.**

| [M+H]$^+$ | $R_t$ / min | proposed structure | MS/MS fragments | *δm/m* / ppm |
|---|---|---|---|---|
| 113.0351 | 2.381 | 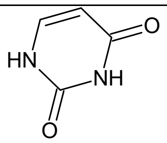 | M$^+$= 70.0293 | 4.83 |
| | | | 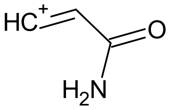 | |
| | | | M$^+$= 96.0086 | |
| | | | 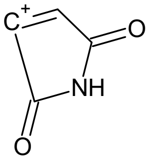 | |
| | | | M$^+$= 43.0187 | |
| | | | 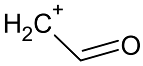 | |
| 121.0720 | 1.309 | 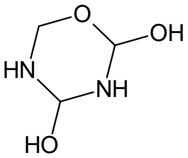 | [M+H]$^+$= 61.0401 | 92.78 |
| | | | 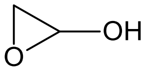 | |
| | | | M$^{*+}$= 44.0134 | |
| | | | 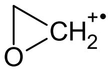 | |
| 293.0311 | 1.497 | n.a. | (202.0611) | n.a. |
| | | | (156.0376) | |
| | | | (111.0214) | |
| | | | (171.0421) | |
| | | | (193.0568) | |
| | | | (221.0339) | |

**Table A. 4 continued**

| $[M+H]^+$ | $R_t$ / min | proposed structure | MS/MS fragments | *δm/m* / ppm |
|---|---|---|---|---|
| 111.0167 | 1.835 | 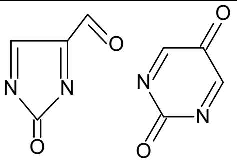<br>molecular formula:<br>$C_4H_2N_2O_2$ | $M^{*+}$= 57.0338<br>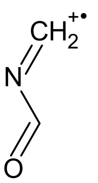<br>$[M+H]^+$= 45.0340<br>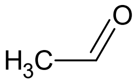<br>$[M+H]^+$= 82.9804<br>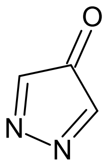 | 19.85 |
| 117.0294 | 1.528 | 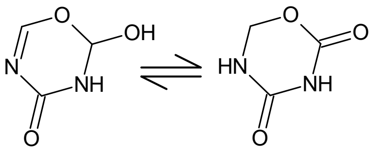 | $M^{*+}$= 58.0655<br>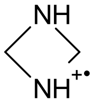 | 0.58 |

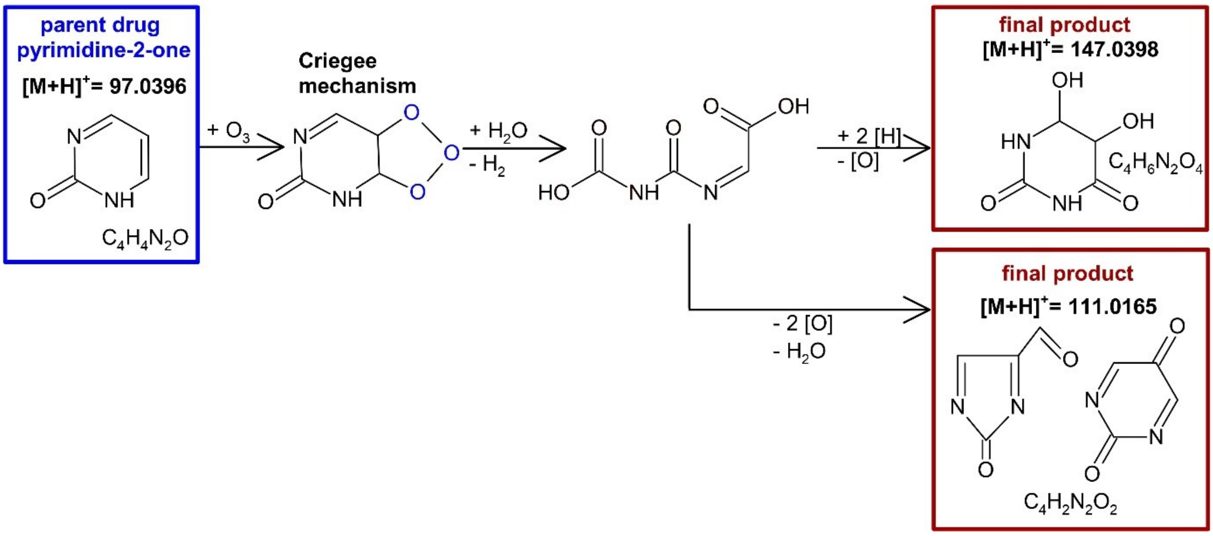


**Figure A. 6: Proposed reaction mechanism of the ozonation of pyrimidine-2-one leading via the intermediate products to the final products *i.e.*, whose *c-t* curves no longer change during the observation period, *cf*. Figure A. 7 yellow squares and blue diamonds.**

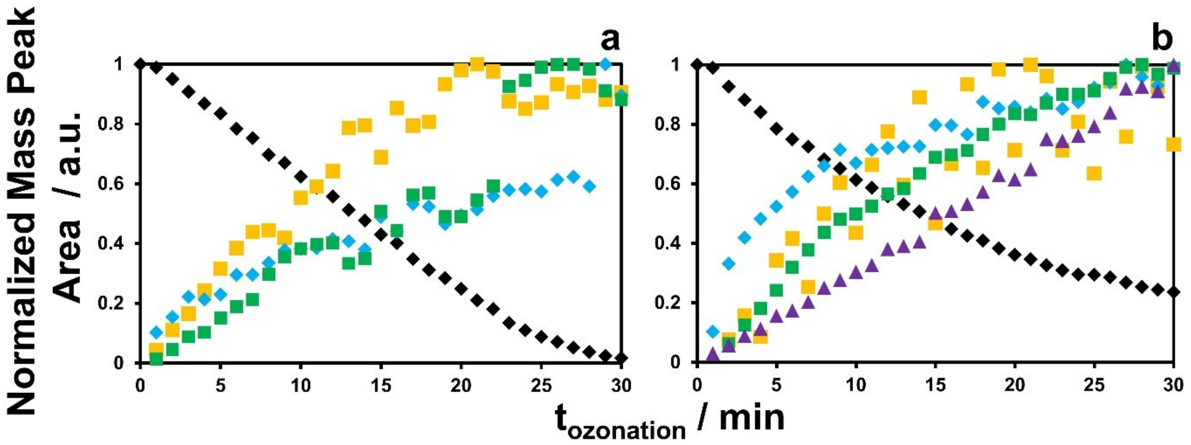


**Figure A. 7: Degradation curves during ozonation of pyrimidine-2-one in deionized $H_2O$ (a) and in +10% BuOH (b) with parent drug pyrimidine-2-one $[M+H]^+$= 97.0396 (black diamonds), and 147.0398 (yellow squares), 111.0165 (blue diamonds), and 230.9814 (green squares) (a,b) and $[M+H]^+$= 190.9994 (purple triangles) formed in 10% tert.-BuOH (b).**

**Table A. 5: Pyrimidine-2(1H)-one ([M+H]$^+$= 97.0396) and its observed transformation products (TPs) formed during ozonation including retention time ($R_t$ / min), MS/MS fragmentation patterns, proposed molecular structures, and mass accuracy ($\delta m/m$ / ppm). TPs without proposed structures were marked as not available (n.a.), as more investigations should be necessary for clarification. *m/z* values without proposed fragment structures are shown in parentheses.**

| [M+H]$^+$ | $R_t$ / min | proposed structure | MS/MS fragments | $\delta m/m$ / ppm |
|---|---|---|---|---|
| 97.0396 | 1.975 | 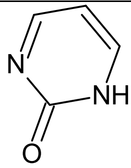 | $M^{+}$ = 79.0711 <br> 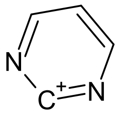 <br> $[M+H]^{+}$ = 61.0350 <br> 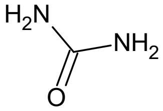 | 0.40 |
| 147.0398 | 1.660 | 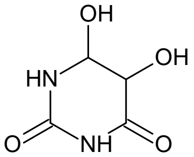 | $M^{*+}$ = 58.0773 <br> 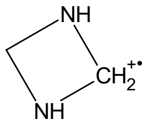 <br> $[M+H]^{+}$ = 86.0600 <br> 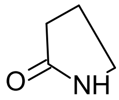 <br> $[M+H]^{+}$ = 101.0558 <br> 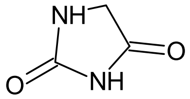 <br> $[M+H]^{+}$ = 71.9750 <br> 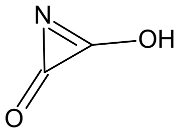 | 1.58 |

**Table A. 5 continued**

| $[M+H]^+$ | $R_t$ / min | proposed structure | MS/MS fragments | $\delta m/m$ / ppm |
|---|---|---|---|---|
| 147.0398 | 1.660 | 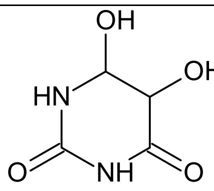 | $[M+H]^+$= 112.9616<br>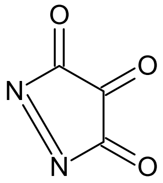 | 1.58 |
| 111.0165 | 1.836 | 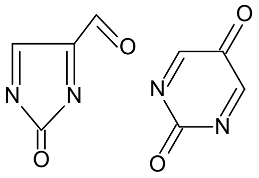<br>molecular formula: $C_4H_2N_2O_2$ | $[M+H]^+$= 71.9976<br>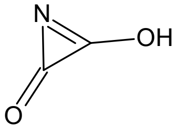<br>(61.0350)<br>$M^+$= 55.1029<br>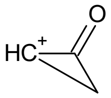<br>(84.0139) | 21.62 |
| 190.9994 | 3.542 | 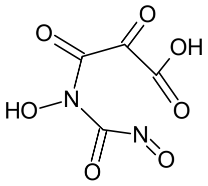 | (84.9964)<br>(138.9595)<br>(128.9975) | 31.01 |
| 230.9672 | 1.859 | n.a. | (116.9762)<br>(142.9480)<br>(101.9839) | n.a. |

**Table A. 6: Molnupiravir ([M+H]$^+$= 330.1363) and its observed transformation products (TPs) formed during ozonation including retention time ($R_t$ / min), MS/MS fragmentation patterns, proposed molecular structures, and mass accuracy ($\delta m/m$ / ppm). TPs without proposed structures were marked as not available (n.a.), as more investigations should be necessary for clarification. *m/z* values without proposed fragment structures are shown in parentheses.**

| [M+H]$^+$ | $R_t$ / min | proposed structure | MS/MS fragments | $\delta m/m$ / ppm |
|---|---|---|---|---|
| 330.1363 | 8.703 | 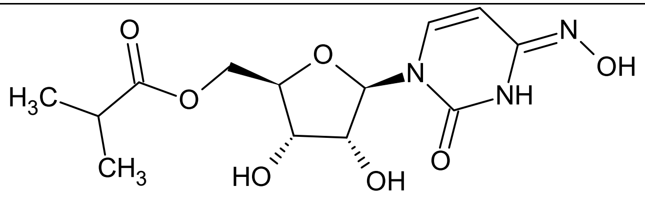 | [M+H]$^+$= 128.0545 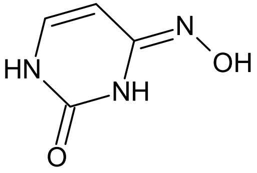 | 20.29 |
| 133.0253 | 1.829 | 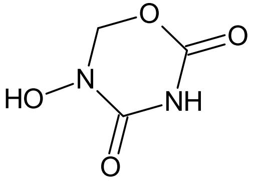 | [M+H]$^+$= 43.0792 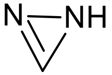 [M+H]$^+$= 61.0427 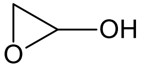 M$^+$= 81.0238 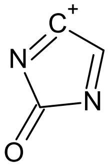 | 6.77 |

**Table A. 6 continued**

| [M+H]⁺ | $R_t$ / min | proposed structure | MS/MS fragments | $\delta m/m$ / ppm |
|---|---|---|---|---|
| 314.1312 | 7.915 | 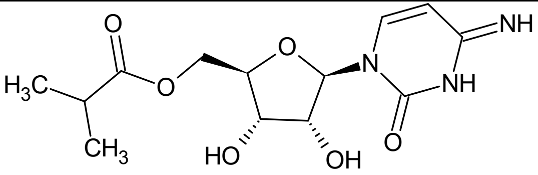 | $[M+H]^+$= 112.0598 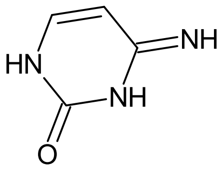 $[M+H]^+$= 103.0613 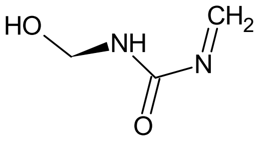 $M^{*+}$= 89.0996 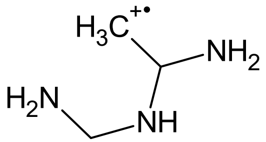 (45.0853) | 11.14 |

**Table A. 6 continued**

| $[M+H]^+$ | $R_t$ / min | proposed structure | MS/MS fragments | $\delta m/m$ / ppm |
|---|---|---|---|---|
| 346.1259 | 7.987 | 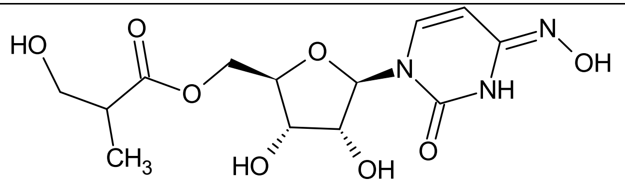 | $M^+$= 43.1041<br>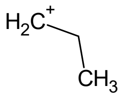<br>$M^+$= 97.0586<br>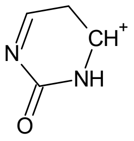<br>$[M+H]^+$= 115.0430<br>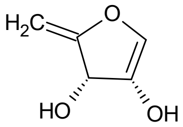<br>$M^+$= 144.0588<br>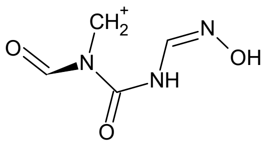 | 4.04 |

**Table A. 6 continued**

| $[M+H]^+$ | $R_t$ / min | proposed structure | MS/MS fragments | $\delta m/m$ / ppm |
|---|---|---|---|---|
| 248.1139 | 8.231 | 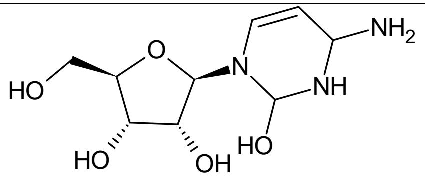 | $M^+$= 43.1045<br>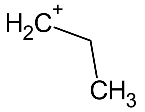<br>$M^+$= 97.0591<br>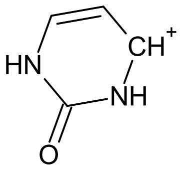<br>$[M+H]^+$= 114.0609<br>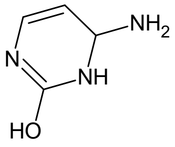 | 41.11 |

**Table A. 6 continued**

| $[M+H]^+$ | $R_t$ / min | proposed structure | MS/MS fragments | $\delta m/m$ / ppm |
|---|---|---|---|---|
| 315.1204 | 8.921 | 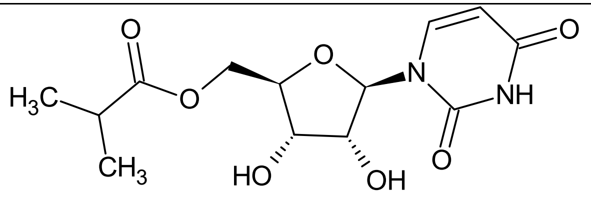 | $M^+$= 43.1043<br>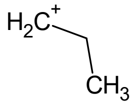<br>$[M+H]^+$= 113.0422<br>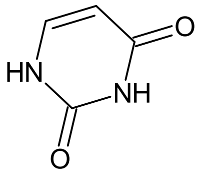<br>$M^+$= 97.0589<br>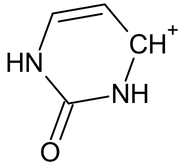 | 5.39 |

 **Table A. 6 continued**

| $[M+H]^+$ | $R_t$ / min | proposed structure | MS/MS fragments | $\delta m/m$ / ppm |
|---|---|---|---|---|
| 559.2288 | 9.612 | n.a. | (220.1520)<br>(357.1428)<br>(203.1260)<br>(155.0795) | n.a. |
| 626.2354 | 10.020 | n.a. | (424.1295)<br>(222.0968)<br>$[M+H]^+$= 112.0597<br>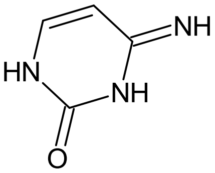 | n.a. |

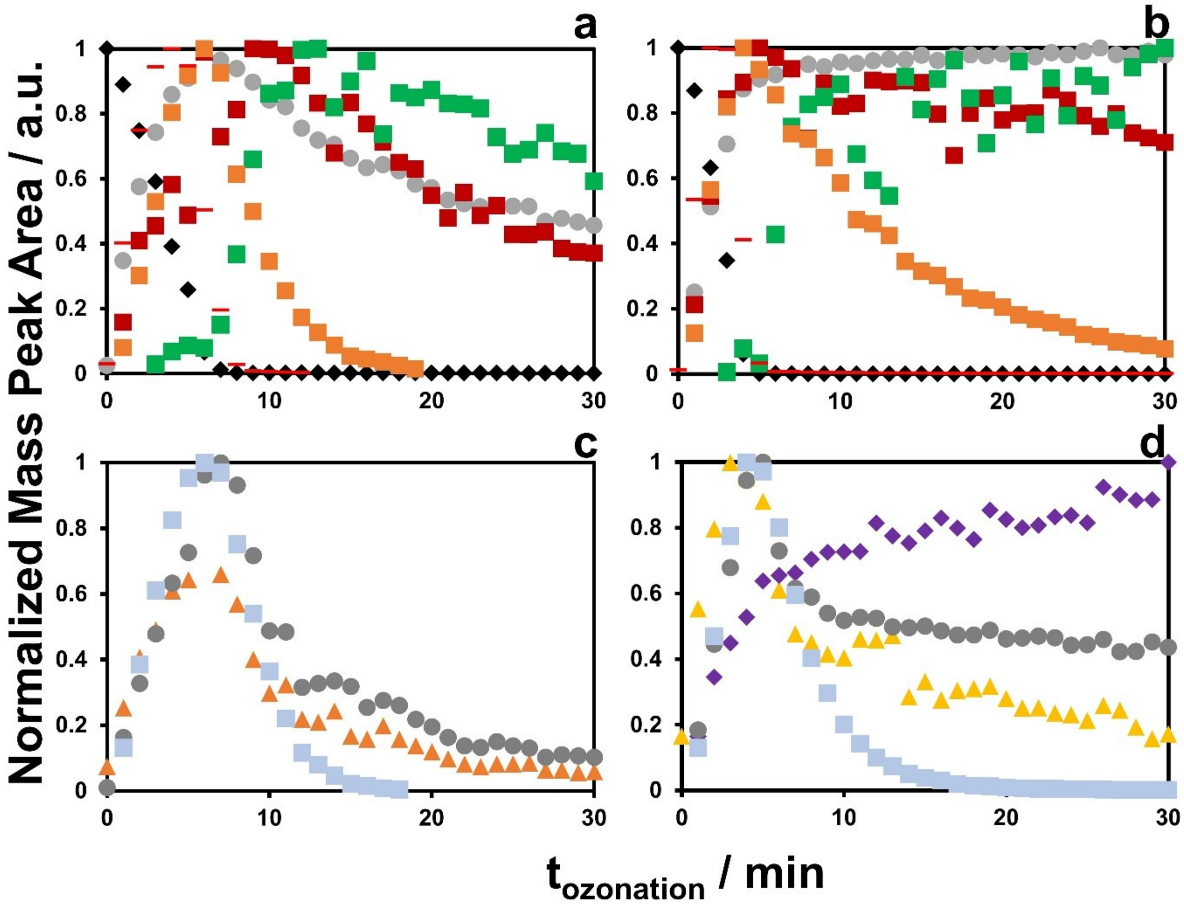


**Figure A. 8: Degradation curves during ozonation of EIDD in deionized $H_2O$ (a,c) and in +10% BuOH (b,d) with parent drug EIDD [M+H]+= 260.0903 (black diamonds), and barely degraded TPs [M+H]+= 200.0397 (gray circles), 246.0727 (red squares), 243.0453 (green squares), 520.1548 (orange squares) and 486. 1739 (red dashes) (a,b); TPs well degraded in water [M+H]+= 244.0789 (orange triangles), 245.0634 (gray circles), and 471.1383 (light blue squares) (c,d); and final TP $[M+H]^+$= 191.0010 only formed in 10% tert.-BuOH.**

**Table A. 7: EIDD-1931 ([M+H]$^+$= 260.0903) and its observed transformation products (TPs) formed during ozonation including retention time ($R_t$ / min), MS/MS fragmentation patterns, proposed molecular structures, and mass accuracy ($\delta m/m$ / ppm). TPs without proposed structures were marked as not available (n.a.), as more investigations should be necessary for clarification. *m/z* values without proposed fragment structures are shown in parentheses.**

| [M+H]$^+$ | $R_t$ / min | proposed structure | MS/MS fragments | $\delta m/m$ / ppm |
|---|---|---|---|---|
| 260.0903 | 3.489 | 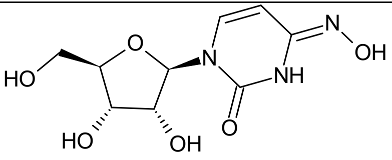 | M$^+$= 128.0440 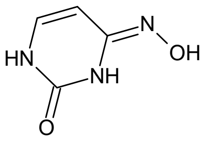 | 9.96 |
| | | | [M+H]$^+$= 82.0800 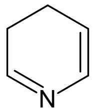 | |
| | | | M$^{*+}$= 110.0461 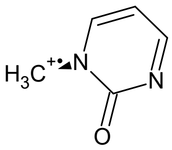 | |
| 200.0537 | 1.521 | 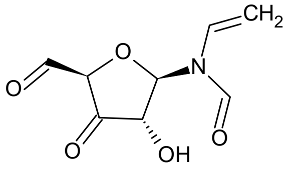 | (97.9938) | 8.00 |
| | | | (56.9905) | |
| | | | (114.9881) | |
| 246.0727 | 1.755 | 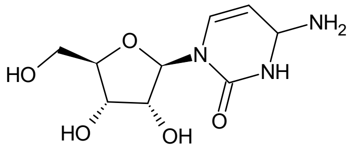 | [M+H]$^+$= 114.0357 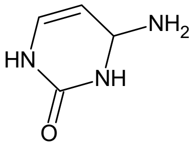 | 145.08 |
| | | | M$^{*+}$= 138.9552 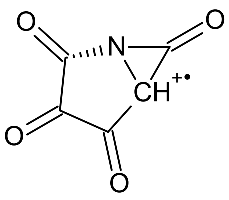 | |

**Table A. 7 continued**

| $[M+H]^+$ | $R_t$ / min | proposed structure | MS/MS fragments | *δm/m* / ppm |
|---|---|---|---|---|
| 246.0727 | 1.755 | 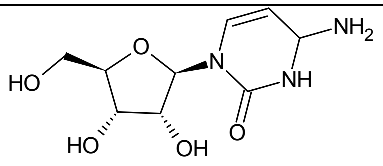 | $M^{*+}$= 57.0818 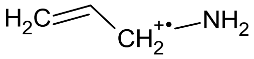 | 145.08 |
| 244.0789 | 2.341 | 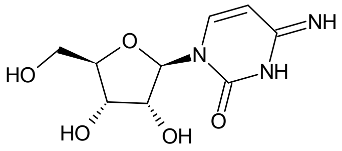 | $M^{*+}$= 112.0590 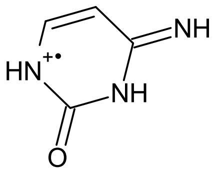 $M^{*+}$= 208.9900 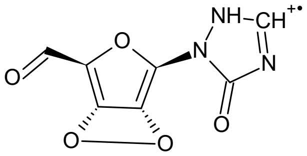 $M^{+}$= 95.0518 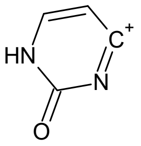 | 56.94 |

**Table A. 7 continued**

| $[M+H]^+$ | $R_t$ / min | proposed structure | MS/MS fragments | $\delta m/m$ / ppm |
|---|---|---|---|---|
| 243.0601 | 2.590 | 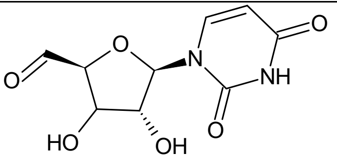 | $M^{*+}$= 114.0356<br>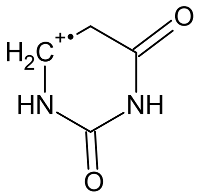<br>(190.9808)<br>$[M+H]^+$= 208.9898<br>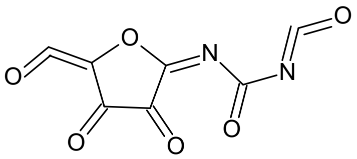 | 4.37 |
| 191.0010 | 3.117 | 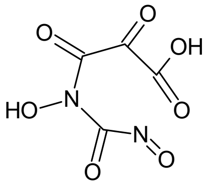 | (84.9973)<br>(138.9593)<br>(135.0406) | 39.39 |
| 245.0776 | 4.810 | 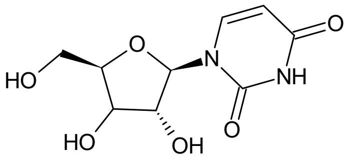 | $[M+H]^+$= 113.0417<br>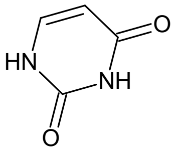<br>$M^{*+}$= 96.0356<br>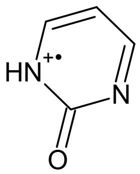<br>$M^{*+}$= 70.0751<br>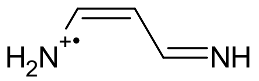 | 3.21 |

**Table A. 7 continued**

| $[M+H]^+$ | $R_t$ / min | proposed structure | MS/MS fragments | $\delta m/m$ / ppm |
|---|---|---|---|---|
| 471.1383 | 6.507 | n.a. | (207.0374)<br>(339.0931) | n.a. |
| 520.1548 | 6.577 | n.a. | $[M+H]^+$= 113.0419<br>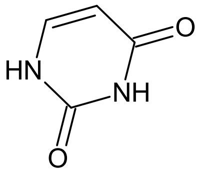<br>(133.0462)<br>(258.0572) | n.a. |
| 486.1492 | 7.421 | n.a. | (222.0481)<br>(354.1082) | n.a. |

**Table A. 8: Overview of ozonation studies on antiviral pharmaceuticals and related structural motifs.**

| Study | Compound(s) | Process / matrix | Analytical approach | Structural elucidation / classification | Relevance relative to present work |
|---|---|---|---|---|---|
| This work | $N^4$-hydroxycytosine, cytosine, uracil, pyrimidine-2-one, molnupiravir, EIDD-1931 | Ozonation in ultrapure water and 10% tert-BuOH | HPLC-ESI-Q-TOF-HRMS with MS/MS | MS/MS-supported structure proposals; confidence classification according to Schymanski et al. (2014) | First ozonation study of molnupiravir and EIDD-1931; model-compound cascade for mechanistic linkage |
| Merkus et al., 2024 [1] | Purine, adenine, guanine and purine-based antiviral motifs | Ozonation at controlled pH | HPLC-Orbital Ion Trap-HRMS | HRMS- and MS/MS-based structure proposals | Structural motif-oriented ozonation of purine analogues |
| Funke et al., 2021 [2] | Zidovudine, thymidine | Ozonation | HPLC-LTQ Orbital Ion Trap-HRMS | MS/MS-based structure proposals | Ozonation of nucleoside Antivirals with pathway discussion |
| Liu et al., 2021 [3] | Ribavirin | Ozonation and ozonation/PMS under laboratory conditions | UV-HPLC, GC–MS | Oxidation induced transformation products identified by MS | Investigation of ozone and radical contributions |

**Table A. 8 continued**

| Study | Compound(s) | Process / matrix | Analytical approach | Structural elucidation / classification | Relevance relative to present work |
|---|---|---|---|---|---|
| Zheng et al., 2024 [4] | Oseltamivir | Ozonation of wastewater effluent | UPLC-TOF-MS | Degradation pathway proposed | Application-oriented antiviral ozonation study |
| Dogruel et al., 2025 [5] | Favipiravir, oseltamivir | Ozonation and catalytic ozonation | UPLC-MS/MS | Transformation product discussion | Evaluation of antiviral removal and ecotoxicological context |
| Prasse et al., 2010 [6] | Acyclovir, carboxy-acyclovir | Ozonation in aqueous solution | HPLC-ESI-Q-TOF-HRMS/MS | Oxidation induced transformation products characterized by MS | Antiviral ozonation kinetic and product study |
| Schlüter-Vorberg et al., 2015 [7] | Acyclovir and related compounds | Ozonation | LC-MS/MS LTQ Orbital Ion Trap-MS | Transformation products identified | Emphasis on transformation-linked toxicity |
| Fedorova et al., 2016 [8] | Amantadine, oseltamivir carboxylate, zanamivir | Ozonation of wastewater effluent | LC-HRMS | HRMS-based product identification | Antiviral ozonation under effluent conditions |

# References


1. Merkus VI, Leupold MS, Rockel SP, Schmidt TC (2024) Ozonation products of purine derivatives, the basic structures of antiviral micropollutants. The Science of the total environment 912:169073. 10.1016/j.scitotenv.2023.169073
2. Funke J, Prasse C, Dietrich C, Ternes TA (2021) Ozonation products of zidovudine and thymidine in oxidative water treatment. Water Research X 11:100090. 10.1016/j.wroa.2021.100090
3. Liu X, Hong Y, Ding S, Jin W, Dong S, Xiao R, Chu W (2021) Transformation of antiviral ribavirin during ozone/PMS intensified disinfection amid COVID-19 pandemic. The Science of the total environment 790:148030. 10.1016/j.scitotenv.2021.148030
4. Zheng M, van Beek SJ, Sánchez-Montes I, Xu B, Gamal El-Din M (2024) Ozonation of the antiviral oseltamivir in wastewater effluent: Matrix effect, oxidation pathway, and toxicity assessment. Journal of Environmental Chemical Engineering 12(6):114297. 10.1016/j.jece.2024.114297
5. Dogruel S, Chavoshi N, Bilgin-Saritas N, Khataee A, Topuz E, Pehlivanoglu E (2025) Degradation and ecotoxicity of favipiravir and oseltamivir in the presence of microplastics during ozonation and catalytic ozonation of synthetic municipal wastewater effluents. Journal of Chemical Technology & Biotechnology 100:955–966. 10.1002/jctb.7831
6. Prasse C, Schlüsener MP, Schulz R, Ternes TA (2010) Antiviral Drugs in Wastewater and Surface Waters: A New Pharmaceutical Class of Environmental Relevance? Environmental science & technology 44(5):1728–1735. 10.1021/es903216p

7. Schlüter-Vorberg L, Prasse C, Ternes TA, Mückter H, Coors A (2015) Toxification by Transformation in Conventional and Advanced Wastewater Treatment: The Antiviral Drug Acyclovir. Environmental Science & Technology Letters 2(12):342–346. 10.1021/acs.estlett.5b00291
8. Fedorova G, Grabic R, Nyhlen J, Järhult JD, Söderström H (2016) Fate of three anti-influenza drugs during ozonation of wastewater effluents - degradation and formation of transformation products. Chemosphere 150:723–730. 10.1016/j.chemosphere.2015.12.051
